# Supplementary material for: Helical structure motifs made searchable for functional peptide design
Source: Nat Commun. 2022 Jan 10;13:102. doi: 10.1038/s41467-021-27655-0 (PMC8748493; doi:10.1038/s41467-021-27655-0)
Supplement: Supplementary file 1 — Supplementary Information [file 41467_2021_27655_MOESM1_ESM.pdf]

SUPPLEMENTARY DATA

for

Helical Structure Motifs Made Searchable for Functional Peptide Design

Cheng-Yu Tsai[1,2,3,¶], Emmanuel O Salawu[1,4,5,¶], Hongchun Li[1,6,7,8,¶], Guan-Yu Lin[9,¶], Ting-Yu Kuo[9,¶], Liyin Voon[1,¶], Adarsh Sharma[1], Kai-Di Hu[1], Yi-Yun Cheng[10], Sobha Sahoo[1], Lutimba Stuart[1], Chih-Wei Chen[1], Yuan-Yu Chang[1,10], Yu-Lin Lu[1], Simai Ke[1], Christopher Llynard D. Ortiz[1,11,12], Bai-Shan Fang[7,13], Chen-Chi Wu[3,14], Chung-Yu Lan[9,15]*, Hua-Wen Fu[9,15]*, Lee-Wei Yang[1,4,16,17]*

[1]Institute of Bioinformatics and Structural Biology, National Tsing Hua University, Hsinchu 300044, Taiwan; [2]Graduate Institute of Medical Genomics and Proteomics, National Taiwan University College of Medicine, Taipei 100025, Taiwan; [3]Department of Otolaryngology, National Taiwan University Hospital, Taipei 100225, Taiwan; [4]Bioinformatics Program, Institute of Information Sciences, Academia Sinica, Taipei 115201, Taiwan; [5]Machine Learning Solutions Lab, Amazon Web Services (AWS), Herndon, VA, USA; [6]Research Center for Computer-Aided Drug Discovery, Shenzhen Institutes of Advanced Technology, Chinese Academy of Sciences, Shenzhen 518055, China; [7]College of Chemistry and Chemical Engineering, Xiamen University, Xiamen 361005, China; [8]Department of Computational and Systems Biology, School of Medicine, University of Pittsburgh, Pittsburgh, PA, 15213, USA; [9]Institute of Molecular and Cellular Biology, National Tsing Hua University, Hsinchu 300044, Taiwan; [10]Praexisio Taiwan Inc. New Taipei 221425, Taiwan; [11]Chemical Biology and Molecular Biophysics Program, Institute of Biological Chemistry, Academia Sinica, Taipei 115201, Taiwan; [12]Department of Chemistry, National Tsing-Hua University, Hsinchu 300044, Taiwan; [13]The Key Laboratory for Chemical Biology of Fujian Province, Key Lab for Synthetic Biotechnology of Xiamen City, Xiamen University, Xiamen 361005, China; [14]Department of Medical Research, National Taiwan University Hospital Hsin-Chu Branch, Hsinchu 302058, Taiwan; [15]Department of Life Science, National Tsing Hua University, Hsinchu 300044, Taiwan; [16]Physics Division, National Center for Theoretical Sciences, Taipei 106319, Taiwan; [17]PhD program in Biomedical Artificial Intelligence, National Tsing Hua University, Hsinchu 300044, Taiwan.

¶ Authors share equal contributions

* To whom correspondence should be addressed: Lee-Wei Yang. Tel: +88635742467; Fax +88635715934; Email: lwyang@life.nthu.edu.tw; Hua-Wen Fu. Tel: +88635742485.
Fax: +88635715934 Email: hwfu@life.nthu.edu.tw, and Chung-Yu Lan. Tel: +88635742473;
Email: cylan@life.nthu.edu.tw

Supplementary Results

Chemical structures of POPC and POPG lipids

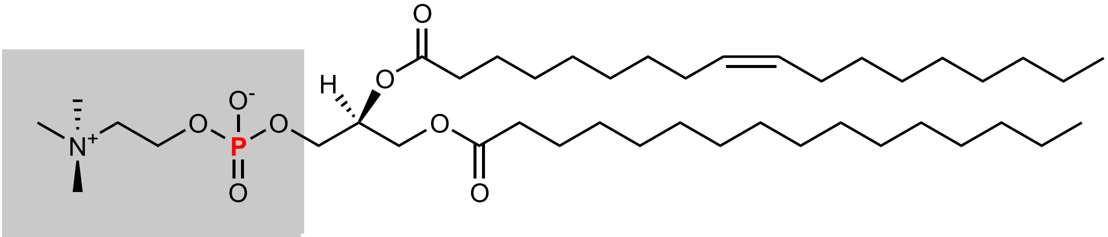

1-palmitoyl-2-oleoyl-glycero-3-phosphocholine, POPC (PC)

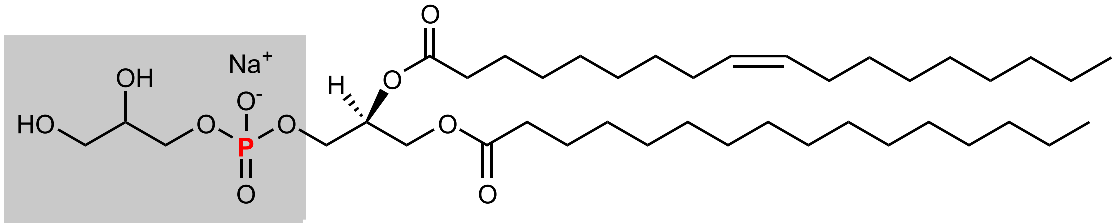

1-palmitoyl-2-oleoyl-sn-glycero-3-phosphoglycerol, POPG (PG)

Supplementary Figure 1. Chemical structures of POPC (PC) and POPG (PG). The regions colored in gray highlight the difference between neutralized and negative-charged head groups in POPC and POPG, respectively. The phosphor atoms of the lipids are highlighted in red, the average position of which in membrane leaflets delineates water-lipid interface. AMP atoms below this interface can be considered as inserted in Figures 3 and Supplementary Fig. 3. Both molecules are generated from ChemDraw (ver. 20.1.1, Perkinelmer Informatics, Inc, MA, USA).

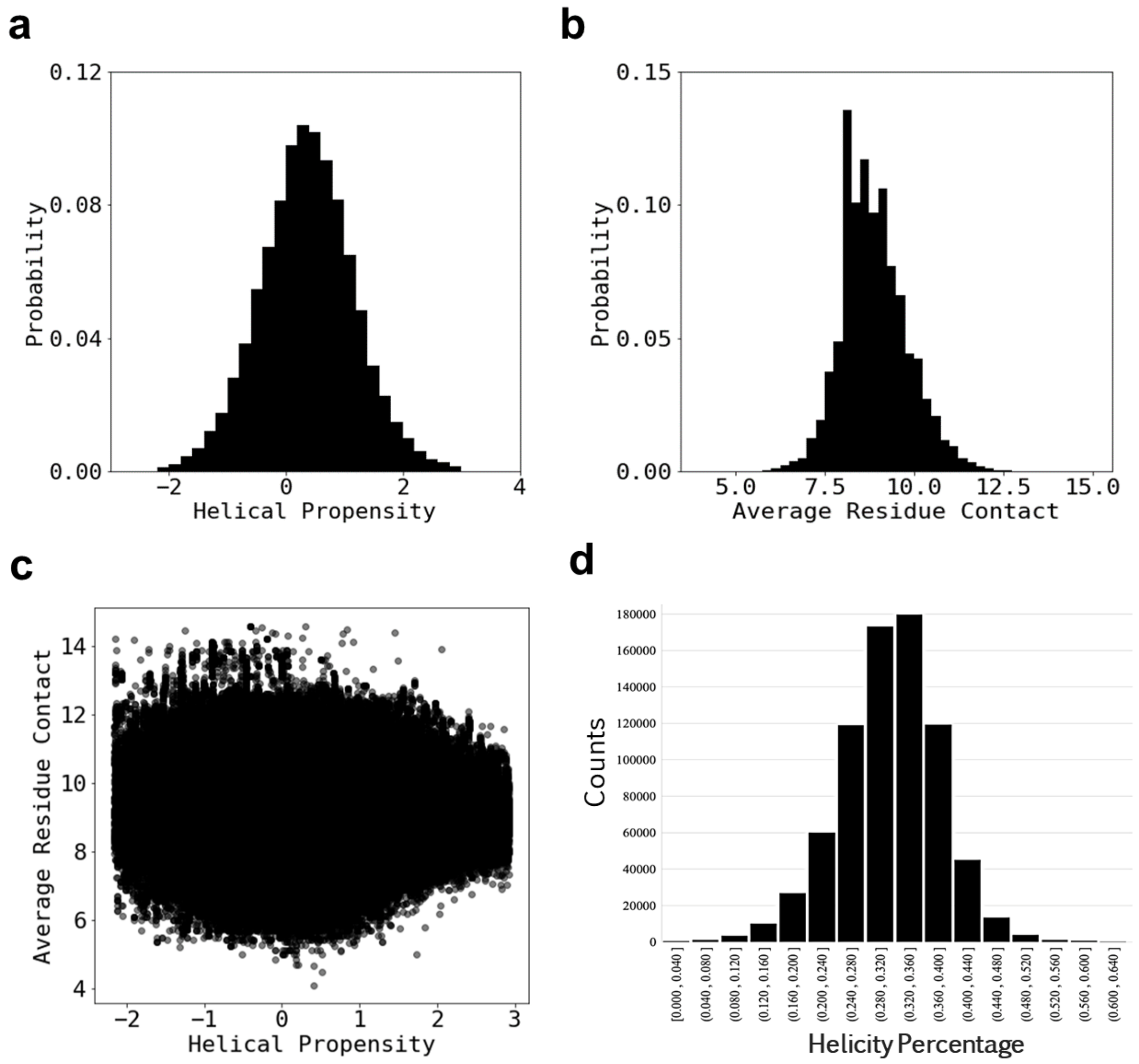

Supplementary Figure 2. Distributions of the helical propensity and the average residue contact for the 1.67 million helical peptides in the TP-DB. (a) Helical propensity is distributed with a mean of 0.38, standard deviation of 0.85 and a most populated bin at 0.20 to 0.40, while the distribution of the average residue contact (b) has a mean of 8.87 and a standard deviation of 0.96, which is most populated at 8.00 to 8.25. A Source Data of both (a) and (b) are provided as a Source Data file. (c) The Pearson correlation between helical propensity and average residue contact is −0.082, suggesting a very weak linear relationship. (d) The estimated helicity % for TP-DB, excluding proline-contained peptides. The estimation is based on the relation Helicity Percentage = 0.597 -0.037 *Avg. Res. Contact + 0.076* log(HP_{NA}), established by MD simulation results of 23 TP-DB peptides (see Fig 6). Nearly half of the database have a helicity% > 32% in isolation. The 1.67 million helical peptides are provided as the Supplementary Data 1 (Supplementary_Data_1.zip) shared in Zenodo repository[1] and source data of the figure panels are provided as a Source Data file.

Natural insertion of three AMPs into bacteria membrane

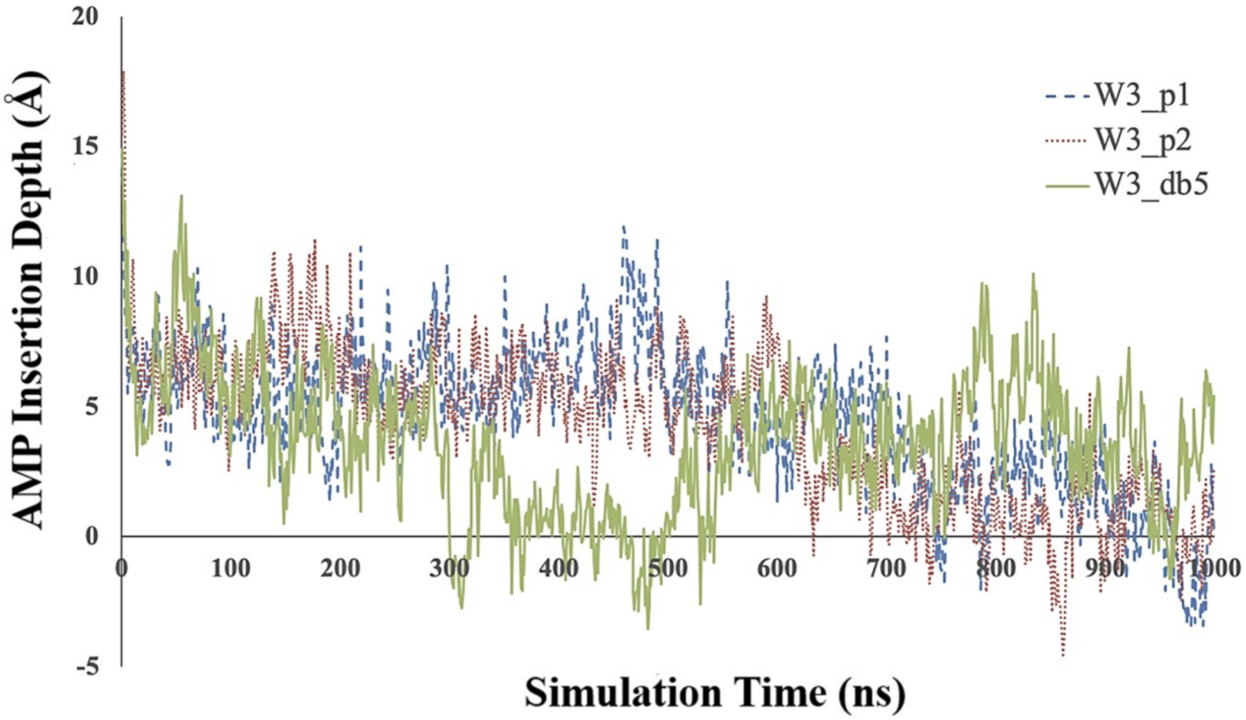

Supplementary Figure 3. The relative position of the center of mass (COM) of AMPs to the COM of phosphor atoms in the upper leaflet consisting of 40 POPC/POPG (3:1) lipid molecules, mimicking bacteria membrane, is plotted against the simulation time. All the MD simulations were conducted for 1 microsecond (us) at body temperature and normal pressure (1 atm) using OpenMM[2] with CHARMM36 forcefield[3,4]. A Source Data of this figure is provided as a Source Data file.

Design of peptide inhibitor against Sgo1-PP2A interaction

Below we demonstrate how TP-DB can be used to design helical blockers to prevent disease-related protein-protein interaction.

In late-stage hepatocellular carcinoma (HCC), patients rely only on targeted therapy and chemotherapy, which were proved generally ineffective. Novel therapeutic targets for treating HCC are urgently needed. Researchers previously found that protein Shugoshin-1 (Sgo1), protecting the stability of centromeric cohesin at centromere and ensuring proper chromosome separation[5], was up-regulated in HCC. Notably, hepatoma cells were found more sensitive to Sgo1 deficiency than normal hepatocytes[6]. These results could suggest that Sgo1 can be a potential therapeutic target for HCC. It has been known that phosphorylated-Sgo1 recruits protein phosphatase 2A (PP2A) to centromere during early mitosis[7] and the Sgo1-PP2A complex sequestered SA2 subunit of cohesin from Plk1-mediated phosphorylation to maintain its stability[8]. Additionally, Sgo1-PP2A complex also maintains Sororin in a hypo- phosphorylated state and keeps the interaction of Sororin-Pds5 to counteract WAPL[7]. These regulations ensure centromeric cohesin tethering sister chromatids together, until the onset of anaphase (Supplementary **Fig.4**). As a result, an inhibitor (possibly a peptide) that blocks the Sgo1-PP2A interaction could potentially suppress the rapidly growing hepatoma cells, susceptible to improper Sgo1-PP2A association, in mitosis.

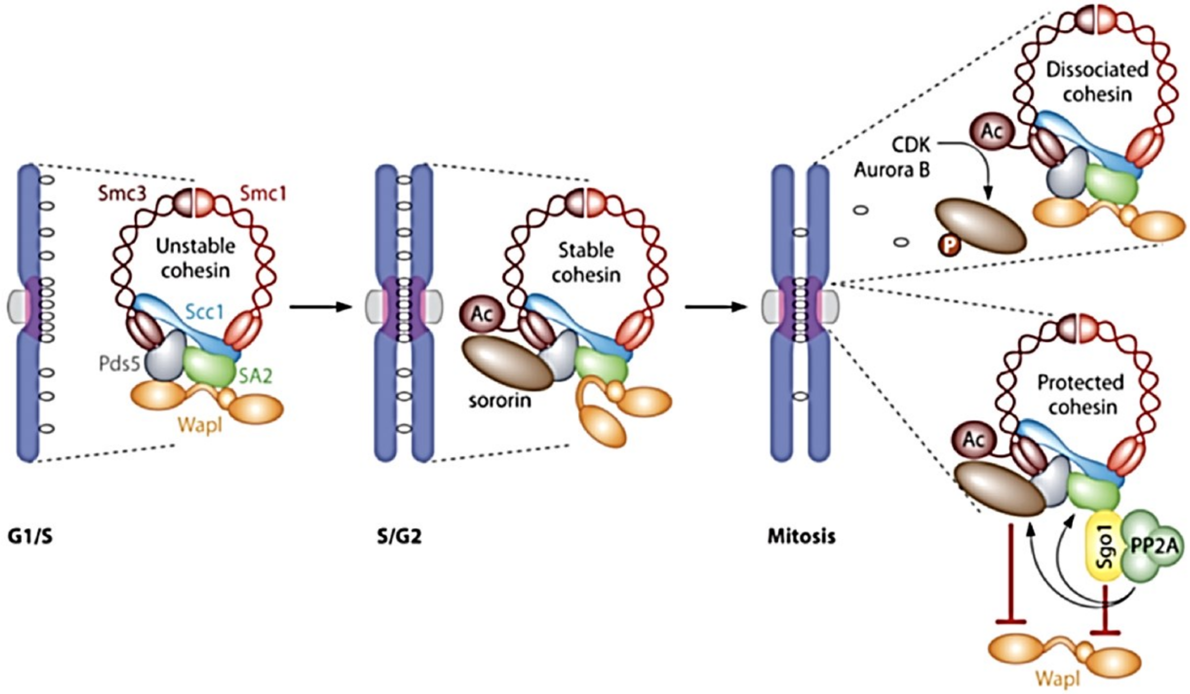

Supplementary Figure 4. The overview of Sgo1-PP2A protecting centromeric cohesin. During mitosis, phosphorylation of SA2 and Sororin cause cohesin to dissociate from the chromosome arm. Cdk1-mediated phospho-Sgo1 recruits PP2A at centromere to counteract phosphorylation of SA2 and Sororin and maintains centromeric cohesin to tether sister chromatids together. Image is adopted from Fig. 3 in Marston's review paper[9] where the permission (Permission for the reuse of Supplementary Figure 4.pdf) is obtained through the Copyright Clearance Center of the publisher (https://marketplace.copyright.com/rs-ui-web/mp).

We noted that the complex of Sgo1-PP2A has been solved by x-ray crystallography (PDB ID: 3FGA) and the two molecules bind with each other through an helix-helix interface (one helix from each molecule)[10]. To design anticancer peptides as blocker against Sgo1-PP2A interaction, the helical stretch in PP2A (residues from 352-392 in chain B of PDB 3FGA), known to form the helix-helix interaction with another helix in Sgo1 (residues from 75 to 92 in chain D of PDB 3FGA) (**Supplementary Figure 5**), is chosen as the candidate target for further investigation. Evidenced from the x-ray structure, the helix-helix interaction is visibly apparent to be the most important essential interacting elements at the protein-protein interface. As shown in **Supplementary Figure 5a**, K374, G378 and Y381 in the helical stretch "KTIHGLIY" could be the anchoring residues on

PP2A to mediate the Sgo1-PP2A interaction, which is supported by our calculation of energetics using *in silico* alanine scanning (**Supplementary Figure 5b**).

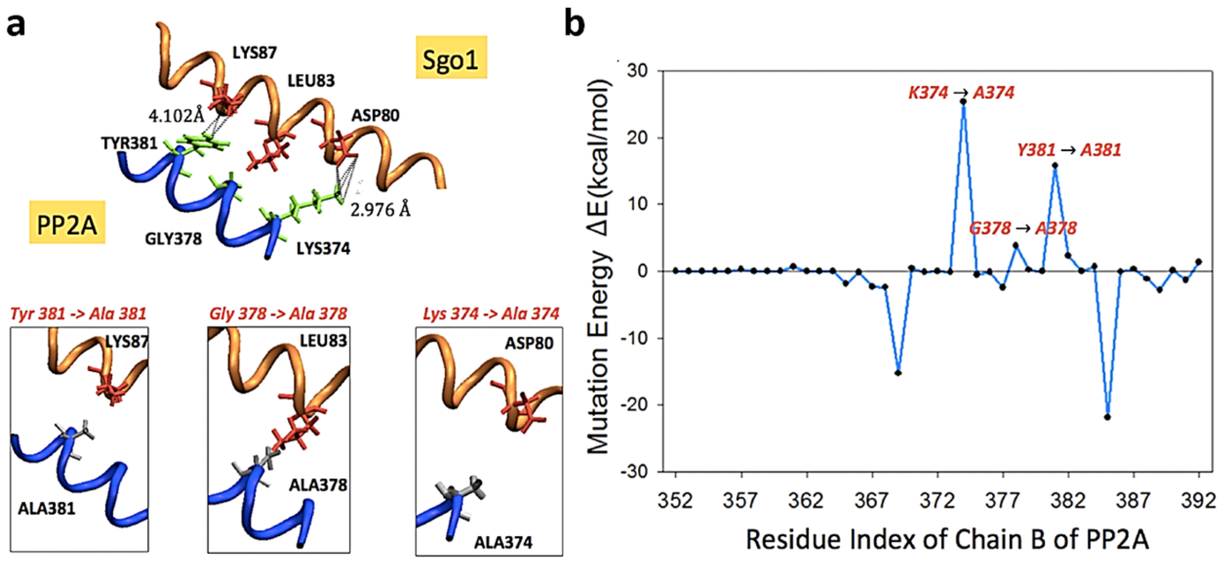

Supplementary Figure 5. Protein-protein interaction (PPI) between Sgo1 and PP2A through helix-helix interactions. Panel **(a, upper)** shows only the PPI interface between Sgo1 and PP2A, while the rest of proteins (3FGA; [10]) are hidden for clarity. Sgo1-PP2A interaction is mediated by two helices, one from Sgo1 (PDB: 3FGA; $V_{75}KEAQDIILQLRKECYYL_{92}$ in chain D) and the other from PP2A (PDB: 3FGA; $K_{369}THMNKTIHGLIYNALK_{385}$ in chain B). Computational alanine scanning for every residue in the PP2A helical fragment (from residue 352 to 392 of chain B) is carried out, and in panel (**b**) energetic contribution for each residue mutated into alanine is assessed (from residue 369 to 385 of chain B) where a large positive value (e.g. for K374A, G378A and Y381A) indicates the importance of the residues in binding, and a large negative value suggests mutation into alanine favors the binding. The non-bonded energy is evaluated by NAMD package[11] using CHARMM 36 forcefield[3,4]. Here in panel **(b)**, the mutation energy for the n-th residue, ΔEn, is defined as $\Delta En = Yn - X0$ where X0 is the non-bonded energy of original complex between Sgo1 and PP2A (for the 352-392 fragment), and Yn is the energy of the complex with the *n*-th residue in PP2A being mutated into alanine, after a short energy minimization. A Source Data of panel (b) is provided as a Source Data file.

To block the protein-protein interaction (PPI) between Sgo1 and PP2A, one could potentially modify part of the most essential interacting element in PP2A into a peptide that interacts with Sgo1 better than its template. As shown in **Supplementary Figure 5b**, computational alanine

scanning helps detecting a stretch from 370 to 384 in PP2A (chain B) dominantly contributing the affinity in the PPI of Sgo1-PP2A complex, especially from K374, G378 and Y381 (i.e. aforementioned essential interacting element in PP2A). We then used this helical stretch, or the extended one with its flanking region from residue 364 to 388, including two energetically favored point mutations, K369A and K385A, as the starting template.

To find the helical peptides that bind the interface helix of Sgo1 stronger than PP2A does, we search the TP-DB for PP2A-like helical peptides using the queries summarized in **Supplementary Table 1**. As detailed below, we first used the pattern "**K***G**Y**" to search TP-DB and discover 69 unique PP2A-like helical peptides (**Supplementary Table 1** and **Supplementary Figure 6**); we then carried out molecular dynamics simulations (MD) to assess how these peptides interact with Sgo1 at the helix-helix interface. Helices H60, H54, H61, H33 and H29 (**Supplementary Figure 7**) are energetically promising for further experimental confirmation. Additionally, we examined other sets of peptides that met the patterns consisting of three anchoring residues ("A***K***G" and "G**Y***A") or consisting of at least four residues ("A***K***G**Y", "K***G**Y***A", "A***K***G**Y***A" and "A**/***K**/***G**/***Y**/***A") in TP-DB. All the corresponding results obtained from the queries are illustrated in **Supplementary Figures 8** to **13**. Among them only the "A***K***G**Y***A" pattern (according to **Supplementary Figure 5b**) returns no result from TP-DB.

Supplementary Table 1. PP2A-like patterns searched against the developed helical peptide database

Pattern	Query	# of Peptides Found	# of Unique Peptides Found
K *** G ** Y	K 3 G 2 Y	398	69
A *** K *** G	A 3 K 3 G	2981	391
G ** Y *** A	G 2 Y 3 A	2079	225
A *** K *** G ** Y	A 3 K 3 G 2 Y	15	3
K *** G ** Y *** A	K 3 G 2 Y 3 A	3	2
A *** K *** G ** Y *** A	A 3 K 3 G 2 Y 3 A	0	0
A**/***K**/***G**/***Y**/***A	A 2,3 K 2,3 G 2,3 Y 2,3 A	34	3

#	U#	Matched Sequence (MS)	Matched Pattern	Full Helix (FH)	PDB ID: Chain	Positions in PDB (MS), (FH) & File Download	Helical Propensity	Contact	Interacting Partners	Helicity%
1	1	KAAEGLKY	K3G2Y	GESLKKAAEGLKYEDFAK	1exe:A	(87,94) (82,99)	0.831	10.250		0.281
2	2	KAAQGAMY	K3G2Y	SRDLRLSLAKAITQRVILKAAQGAMY	1t3q:C	(278,285) (260,285)	0.829	8.750		0.336
4	3	KNAWGEAY	K3G2Y	SPEMKNAWGEAYDQLVAAIKS	3qqq:B	(139,146) (135,155)	0.745	9.750		0.293
6	4	KERLGEAY	K3G2Y	QESAVANTKERLGEAYQA	4poo:A	(62,69) (54,71)	0.744	7.375		0.381
7	5	KVAWGQAY	K3G2Y	SPEMKVAWGQAYDHLVAAIKAEMNLS	3zhw:B	(138,145) (134,159)	0.624	9.625		0.288
9	6	KLQLGQLY	K3G2Y	TDTEVERAKSLLKLQLGQLYE	3cxh:A	(371,378) (359,379)	0.583	8.875		0.313
20	7	KQKLGKAY	K3G2Y	KQKLGKAYFQVQKIEAELYQLIKVSH	1skv:D	(39,46) (39,64)	0.564	7.500		0.362
24	8	KEQLGEFY	K3G2Y	TKEQLGEFYEALDCL	3apw:B	(135,142) (134,148)	0.522	8.125		0.336
31	9	KRKMGEAY	K3G2Y	LHDSASKECKRKMGEAYALNKKM	3fy4:C	(490,497) (481,503)	0.519	8.875		0.308
34	10	KMYLGYEY	K3G2Y	DAKMYLGYEYVTAIRNLRE	3fgs:A	(312,319) (310,328)	0.502	7.375		0.362
84	11	KTLAGQLY	K3G2Y	NKTLAGQLYSELKEFF	1d9z:A	(67,74) (66,81)	0.464	9.000		0.299
94	12	KQQMGKEY	K3G2Y	KQQMGKEYREKIEAELQDICNDVLELLDKY LIPNA	2bq0:B	(77,84) (77,111)	0.448	8.375		0.321
97	13	KLMKGQLY	K3G2Y	CRFVKLMKGQLYIDTVAA	1q4q:H	(294,301) (290,307)	0.443	7.375		0.358
109	14	KERRGEMY	K3G2Y	GNLKERRGEMYHYFYDQLLTRYYFERLTN	3gwj:D	(243,250) (240,268)	0.432	9.625	3gwj:D (347, 358)<br>3gwj:D (359, 363)<br>3gwj:D (374, 385)<br>3gwj:D (409, 428)<br>3gwj:D (533, 535)<br>3gwj:D (595, 597)	0.274
112	15	KNAWGVAY	K3G2Y	SPEMKNAWGVAYDQLVAAIKFE	3qqr:B	(139,146) (135,156)	0.422	9.500		0.278
114	16	KMLLGATY	K3G2Y	KMLLGATYFLPIYATVFIVGGFWEVLFCMV R	4p6v:B	(121,128) (121,151)	0.404	11.375		0.207
115	17	KASAGKLY	K3G2Y	SKASAGKLYEAG	1p0n:B	(192,199) (191,202)	0.393	8.625	1p0n:B (237, 249)<br>1p0n:B (329, 339)	0.308
119	18	KSEWGRAY	K3G2Y	GVLEGLKVKSEWGRAYG	1x9f:J	(13,20) (5,21)	0.381	8.625	1x9f:J (58, 78)<br>1x9f:J (102, 122)<br>1x9f:J (126, 143)<br>1x9f:K (27, 47)	0.307
125	19	KHEKGREY	K3G2Y	LEEILEKHEKGREYG	2cxi:A	(182,189) (176,190)	0.355	8.250		0.319
128	20	KEFLGDEY	K3G2Y	YKEFLGDEYYASVASLNTC	1aov:A	(654,661) (653,671)	0.337	8.125		0.322
⋮										
347	56	KTIHGLIY	K3G2Y	KTIHGLIYNALKLFME	2iae:B	(384,391) (384,399)	-0.200	8.750		0.258
349	57	KWGMGQHY	K3G2Y	KWGMGQHYELGSYIRRRYGRFL	1rpt:A	(40,47) (40,61)	-0.215	10.125	1rpt:A (78, 91)	0.206
351	58	KKIVGESY	K3G2Y	LDSERDKARKEVEEYVKKIVGESYAKS	2m6u:A	(17,24) (1,27)	-0.241	8.250	2m6u:A (58, 81)	0.273
352	59	KDILGHVY	K3G2Y	SKDILGHVYEYFLGQFALAEG	3khk:B	(201,208) (200,220)	-0.243	8.500	3khk:B (255, 267)<br>3khk:B (291, 305)	0.264
353	60	KSCYGKKY	K3G2Y	KSCYGKKYG	1b8t:A	(59,66) (59,67)	-0.252	7.625		0.296
354	61	KFDKGYSY	K3G2Y	DPDKFDKGYSYNIRHSF	3q36:B	(340,347) (337,353)	-0.279	13.250		0.086
356	62	KGTAGYIY	K3G2Y	SWDDWDYAAKVTLANSQKGTAGYIYRFLHD VSEG	2wxt:A	(230,237) (213,246)	-0.354	9.500		0.219
368	63	KDIIGICY	K3G2Y	LRWLAKSFFELTVLKDIIGICY	3phf:U	(329,336) (315,336)	-0.462	61.750	3phf:U (368, 383)<br>3phf:U (415, 434)	-1.723
384	64	KKPKGRAY	K3G2Y	TEKPKKPKGRAYKRL	3izb:Z	(28,35) (24,38)	-0.492			
385	65	KASLGTGY	K3G2Y	DTAEALATAKASLGTGYKKVSKSYTGEWKK YLNSL	1ulv:A	(246,253) (237,271)	-0.496	8.750	1ulv:A (330, 345)<br>1ulv:A (346, 361)	0.236
387	66	KPEKGVQY	K3G2Y	DVIRKRHYRIGLNLFNKKPEKGVQYLIER	4c0a:F	(416,423) (399,427)	-0.676	9.250	4c0a:F (448, 457)<br>4c0a:F (461, 474)<br>4c0a:F (595, 607)<br>4c0a:F (724, 757)	0.203
391	67	KTYLGPQY	K3G2Y	TYKTYLGPQYLTLMDN	3mc2:D	(650,657) (648,663)	-0.677	7.625		0.263
395	68	KPKRGIQY	K3G2Y	QQKEIIEQGIDLFNKKPKRGIQYLQEQ	3ltl:A	(712,719) (697,723)	-0.751	9.375	3ltl:A (744, 753)<br>3ltl:B (700, 723)	0.193
397	69	KGTSGYIY	K3G2Y	SWDEWDYAAKVALANSQKGTSGYIYRFLHD VSD	1kho:B	(230,237) (213,245)	-0.849	9.625		0.176

Supplementary Figure 6. 69 unique helical sequences obtained from the TP-DB matching the pattern K*G**Y given the query of [K 3 G 2 Y].** All the matched patterns are colored in blue, and the residues in bold mean the anchoring residues of matched pattern assumed to mediate the Sgo1-PP2A interaction. The result page is available online at https://dyn.life.nthu.edu.tw/design/result?JobID=612ac7c3p

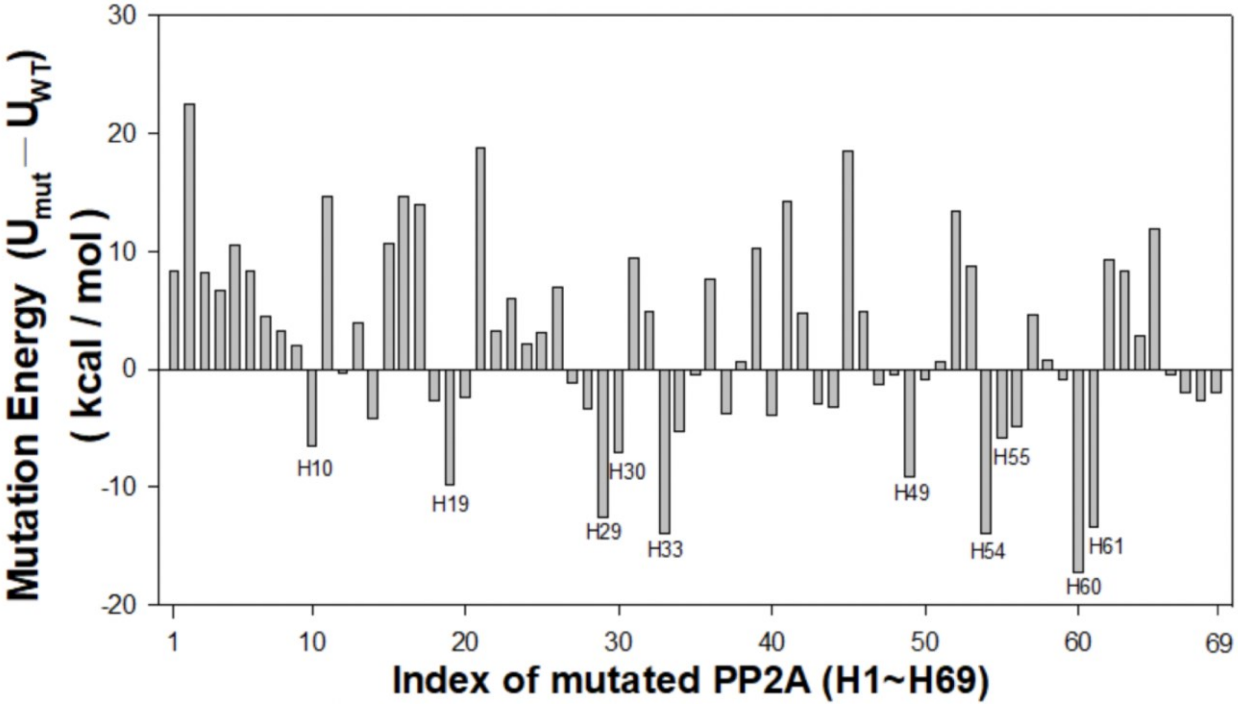

Supplementary Figure 7. Interactions between Sgo1 helix and PP2A-like peptides. U_{mut} is the interaction energy between targeted Sgo1 helix and each of the 69 PP2A-like peptides (from H1 to H69) that match the pattern "K3G2Y". In detail, the energy is calculated by replacing the residue T375, I376, H377, L379 and I380 in the "$\mathbf{K_{374}}$TIH**G**LI$\mathbf{Y_{381}}$" stretch of PP2A with the corresponding residues in each of the 69 pattern-matched (K3G2Y) helical peptides returned from TP-DB. U_{WT} is the interaction energy between Sgo1 helix and PP2A. Larger difference of interaction energies (U_{mut} - U_{WT}) suggest stronger binding of PP2A-like peptides with the Sgo1 helix than that between PP2A and the Sgo1 helix. Top ten values are further labeled in the graph. The Source Data of this figure is provided as a Source Data file.

#	U#	Matched Sequence (MS)	Matched Pattern	Full Helix (FH)	PDB ID: Chain	Positions in PDB (MS), (FH) & File Download	Helical Propensity	Contact	Interacting Partners	Helicity%
1	1	AELAKAEAG	A3K3G	DHAAELAKAEAGVAEMAAKR	3pij:B	(19,27) (16,35)	1.389	8.889	3pij:B (326, 331)	0.374
5	2	AYLAKEAWG	A3K3G	DSDAIGSSVAFAYLAKEAWG	2enx:A	(24,32) (13,32)	1.264	8.778	2enx:A (44, 54)<br>2enx:A (121, 132)<br>2enx:A (137, 153)	0.368
6	3	ALAKKAAEG	A3K3G	GPSGGAAVKALAKKAAEG	3vsd:A	(348,356) (339,356)	1.264	9.444	3vsd:A (370, 373)<br>3vsd:A (374, 382)	0.344
10	4	AAAAKAMKG	A3K3G	GEHVRDALLFAALAAAAKAMKG	1ua4:A	(368,376) (355,376)	1.221	10.556	1ua4:A (388, 390)<br>1ua4:A (437, 453)	0.299
14	5	AAALKWLKG	A3K3G	SGAFLEQIVAIVKLKGEAAALKWLKGLKEY<br>G	3tyh:F	(156,164) (139,169)	1.192	9.444	3tyh:F (220, 223)<br>3tyh:F (292, 306)	0.338
51	6	ARAEKKAAG	A3K3G	KQEKKQRLLARAEKKAAG	3j3b:G	(116,124) (107,124)	1.153			
52	7	ALAEKMALG	A3K3G	APQLSKIMWALAEKMALG	3q12:D	(210,218) (201,218)	1.143	7.778		0.396
54	8	ALARKALEG	A3K3G	ALARKALEG	2dsj:B	(287,295) (287,295)	1.132	8.889		0.354
56	9	AAFAKQAEG	A3K3G	AAFAKQAEGT	2lsh:A	(16,24) (16,25)	1.113	9.111	2lsh:A (70, 74)	0.344
57	10	AEAAKNALG	A3K3G	SYKVIVAEAAKNALG	3gyx:G	(175,183) (169,183)	1.103	9.667	3gyx:G (470, 493)	0.323
63	11	AAREKIAAG	A3K3G	SVIAAREKIAAG	1f76:D	(303,311) (300,311)	1.036	54.889		-1.355
65	12	AYEMKEALG	A3K3G	GGMPAAYEMKEALG	3t31:A	(17,25) (12,25)	1.032	9.556	3t31:A (62, 68)<br>3t31:A (321, 340)<br>3t31:A (385, 405)	0.322
81	13	AKLAKEQLG	A3K3G	GNAGYYTAKLAKEQLG	1euz:F	(228,236) (221,236)	1.020	9.444	1euz:F (256, 268)	0.325
83	14	AALDKELAG	A3K3G	SDEAKAAAAESVKNHLAALDKELAG	1f2e:D	(134,142) (118,142)	1.018	8.444	1f2e:D (175, 187)	0.362
85	15	AAAIKAFAG	A3K3G	EAAAIKAFAGEFD	3exz:E	(36,44) (35,47)	1.013	9.000		0.341
90	16	ADAEKAYEG	A3K3G	GADAEKAYEGFLAFKDVVKKS	1ppr:O	(130,138) (129,149)	1.010	9.111		0.337
98	17	AQLEKELQG	A3K3G	MEELQAQLEKELQGG	3rde:B	(263,271) (258,272)	1.003	8.222		0.369
102	18	AEELKALIG	A3K3G	EAEELKALIGS	4wsr:F	(98,106) (97,107)	0.987	8.667		0.351
114	19	AAFRKAEEG	A3K3G	DLVAAFRKAEEG	3p0b:A	(126,134) (123,134)	0.985	8.667	3p0b:A (154, 174)	0.351
116	20	ADELKEEWG	A3K3G	EIPDIMFKLKTQPNKIADELKEEWG	3t7z:A	(52,60) (36,60)	0.979	7.889		0.380
117	21	ALRLKEELG	A3K3G	RRMLAAREEALRLKEELG	2efy:B	(127,135) (118,135)	0.972	8.444		0.358
122	22	AEAAKMAVG	A3K3G	SYKPIIAEAAKMAVG	2fje:C	(2158,2166) (2152,2166)	0.967	10.222	2fje:C (2447, 2471)	0.292

:

#	U#	Matched Sequence (MS)	Matched Pattern	Full Helix (FH)	PDB ID: Chain	Positions in PDB (MS), (FH) & File Download	Helical Propensity	Contact	Interacting Partners	Helicity%
2801	370	AHGIKVLHG	A3K3G	NANVAAHGIKVLHG	2h8f:D	(62,70) (57,70)	-0.502	8.778	2h8f:D (100, 119)	0.234
2829	371	APAYKKDTG	A3K3G	TSTDNTGLLDVLAPAYKKDTG	3kn3:C	(22,30) (10,30)	-0.509	8.111	3kn3:C (121, 136)<br>3kn3:C (214, 226)	0.258
2832	372	APYLKVFLG	A3K3G	LIAPYLKVFLGFI	1t5b:B	(154,162) (152,164)	-0.514	8.889		0.229
2834	373	APTVKAFAG	A3K3G	APTVKAFAGLLREG	1wlo:A	(72,80) (72,85)	-0.518	9.444	1wlo:A (107, 135)	0.208
2835	374	AGVVKFKKG	A3K3G	DAGVVKFKKG	1lrz:A	(377,385) (376,385)	-0.521	8.667		0.237
2836	375	APFDKYLRG	A3K3G	APFDKYLRG	1rz5:A	(168,176) (168,176)	-0.571	9.778	1rz5:A (285, 298)	0.192
2837	376	AAHGKVVCG	A3K3G	PKVAAHGKVVCGALDKAVK	1ouu:B	(61,69) (58,76)	-0.582	9.444	1ouu:B (124, 144)	0.203
2840	377	AHGVKVLHG	A3K3G	TPDAIKGNAKIAAHGVKVLHG	1v4w:D	(62,70) (50,70)	-0.583	9.000	1v4w:D (100, 119)	0.220
2844	378	APILKLIHG	A3K3G	SAPILKLIHGDLENQFNE	3qhq:A	(74,82) (73,90)	-0.594	8.111	3qhq:A (126, 135)<br>3qhq:A (144, 159)	0.252
2846	379	AVFEKMMPG	A3K3G	PKLITDGLAVFEKMMPGYVSVLESNLT	2a0b:A	(685,693) (677,703)	-0.615	7.667	2a0b:A (744, 773)	0.267
2850	380	AFNAKPKNG	A3K3G	HPEIEKAQREIIEAFNAKPKNGINKIKEIC<br>EQY	1xt0:B	(15,23) (2,34)	-0.644	9.333	1xt0:B (50, 53)<br>1xt0:B (54, 63)<br>1xt0:B (66, 79)	0.203
2855	381	AHHGKVVMG	A3K3G	NPKVAHHGKVVMGGLERAIKN	3bcq:D	(61,69) (57,77)	-0.682	9.778	3bcq:D (100, 119)<br>3bcq:D (124, 143)	0.183
2857	382	AHGKKVAGG	A3K3G	SAQVKAHGKKVAGGLSEAANH	2zfb:A	(57,65) (52,72)	-0.683	9.556	2zfb:A (95, 113)<br>2zfb:A (118, 136)	0.192
2858	383	AILGKVLGG	A3K3G	PVVLVAGTVVTVIAILGKVLGGFLGA	4bwz:A	(301,309) (288,313)	-0.695	11.778	4bwz:A (331, 345)<br>4bwz:A (349, 384)	0.108
2859	384	APYLKTRVG	A3K3G	AAPYLKTRVG	4efz:A	(77,85) (76,85)	-0.695	18.222		-0.130
2861	385	ADPVKAWSG	A3K3G	DEFLKKNPEKVRKFLKAIKKATDYVLADPVKAWSGYIDFK	4h67:H	(233,241) (207,246)	-0.710	7.667		0.259
2871	386	ASVGKMIDG	A3K3G	ATEIRASVGKMIDGIGRFYIQMCTEL	4dyt:C	(27,35) (22,47)	-0.806	10.444	4dyt:C (112, 122)<br>4dyt:C (277, 285)	0.149
2933	387	AHGKKVMGG	A3K3G	SPNIKAHGKKVMGGIALAVSK	3ng6:C	(58,66) (53,73)	-0.884	9.222	3ng6:C (119, 138)	0.189
2966	388	ANTGKSCKG	A3K3G	DQNNIKQAFANTGKSCKGCHDVYK	4ulv:B	(112,120) (103,126)	-0.898	8.667		0.208
2970	389	AHSIKGGAG	A3K3G	AEQLNAIFRAAHSIKGGAGTF	1i5n:B	(47,55) (37,57)	-0.915	9.667		0.170
2975	390	ANSQKGTSG	A3K3G	SWDEWDYAAKVALANSQKGTSGYIYRFLHD<br>VSD	1kho:A	(226,234) (213,245)	-0.943	10.444		0.139
2977	391	APTVKASCG	A3K3G	EAPTVKASCGF	3zjy:E	(831,839) (830,840)	-1.130	9.000		0.178

Supplementary Figure 8. 391 unique helical sequences obtained from the TP-DB for the pattern A*K***G given the query of [A 3 K 3 G].** All the matched patterns are colored in blue, and the residues in bold mean the anchoring residues of matched pattern assumed to mediate the Sgo1-PP2A interaction. The result page is available online at https://dyn.life.nthu.edu.tw/design/result?JobID=612ac8b1z

#	U#	Matched Sequence (MS)	Matched Pattern	Full Helix (FH)	PDB ID: Chain	Positions in PDB (MS), (FH) & File Download	Helical Propensity	Contact	Interacting Partners	Helicity%
1	1	GAAYAEIA	G2Y3A	PGAAYAEIALAAATDTF	4p7p:A	(969,976) (968,984)	0.998	9.250	4p7p:A (1117, 1121)<br>4p7p:A (1124, 1134)	0.331
2	2	GEQYAELA	G2Y3A	TFIAAQFVLYGEQYAELAARR	3s4c:A	(529,536) (519,539)	0.997	10.000	3s4c:A (592, 601)<br>3s4c:A (666, 678)<br>3s4c:A (732, 744)	0.303
12	3	GANYAAWA	G2Y3A	GANYAAWAVNVAQV	1mdt:B	(275,282) (275,288)	0.985	10.000	1mdt:B (310, 314)<br>1mdt:B (326, 347)<br>1mdt:B (358, 376)	0.302
24	4	GARYEALA	G2Y3A	AGARYEALATEIDRGLRFMSACG	2ypq:A	(211,218) (210,232)	0.983	7.625		0.390
39	5	GAEYQRAA	G2Y3A	SAGAEYQRAALISALQTLY	3c0k:A	(132,139) (130,148)	0.944	9.000	3c0k:A (160, 165)	0.336
41	6	GLLYRAAA	G2Y3A	SGLLYRAAAFLALRAG	3w90:A	(34,41) (33,48)	0.939	9.625	3w90:A (85, 88)<br>3w90:A (90, 102)<br>3w90:A (103, 115)	0.312
46	7	GMAYLEEA	G2Y3A	AAETLLGMCLDVCEGMAYLEEA	4rfm:A	(469,476) (455,476)	0.937	9.125	4rfm:A (536, 553)<br>4rfm:A (604, 618)	0.331
102	8	GQAYKLWA	G2Y3A	TKEDLKRRGQAYKLWADQN	2yym:A	(84,91) (76,94)	0.902	10.250	2yym:A (267, 310)<br>2yym:A (315, 342)	0.286
108	9	GAVYLAAA	G2Y3A	GAVYLAAALAAHSQ	1fui:C	(117,124) (117,130)	0.873	11.625	1fui:C (565, 570)	0.233
114	10	GEAYQAIA	G2Y3A	TPEIINAWGEAYQAIADIFITVEKKMYEEA	4g1v:A	(123,130) (115,144)	0.857	8.375		0.352
119	11	GLAYMAMA	G2Y3A	PQYEYLVAMFIPIWSGLAYMAMAI	4t13:A	(48,55) (33,56)	0.850	10.000	4t13:A (69, 92)<br>4t13:A (194, 226)	0.292
125	12	GEEYFALA	G2Y3A	APDGEEYFALALFFASHRWG	3a3v:A	(129,136) (126,145)	0.846	9.875	3a3v:A (195, 199)<br>3a3v:A (200, 211)<br>3a3v:A (265, 279)<br>3a3v:A (357, 373)	0.296
133	13	GAEYATLA	G2Y3A	VFIAAQFVLYGAEYATLAERR	2cqt:B	(529,536) (519,539)	0.833	9.750	2cqt:B (592, 601)<br>2cqt:B (667, 679)<br>2cqt:B (732, 744)	0.300
149	14	GLEYLREA	G2Y3A	GEKGLEYLREAADKY	3pg8:B	(146,153) (143,157)	0.824	9.625	3pg8:B (170, 178)	0.303
165	15	GAVYAELA	G2Y3A	DNYERDAELQQRTGAVYAELAAQG	1mrs:A	(176,183) (163,186)	0.816	9.500		0.308
188	16	GAHYYWEA	G2Y3A	GAHYYWEA	4tkx:L	(577,584) (577,584)	0.807	8.625		0.339
190	17	GAQYIQAA	G2Y3A	GAQYIQAAGVALGLKMR	3duf:G	(145,152) (145,161)	0.772	12.125	3duf:G (178, 191)<br>3duf:H (58, 72)<br>3duf:H (89, 95)	0.207
					:					
1947	207	GPYYFYRA	G2Y3A	GPYYFYRAAVRLGVAAFVFSEAARRDRRAS<br>A	2xqy:E	(262,269) (262,292)	-0.548	9.125		0.218
1949	208	GMGYAIGA	G2Y3A	IGMGYAIGASVTSG	2q28:B	(426,433) (425,438)	-0.563	11.625	2q28:B (453, 455)<br>2q28:B (456, 463)<br>2q28:B (518, 533)	0.124
1955	209	GSFYGRRA	G2Y3A	GTSLGDGSFYGRRAGISAAK	4at2:A	(475,482) (469,488)	-0.567	12.500		0.091
1957	210	GPFYATKA	G2Y3A	YGPFYATKAAIEKAG	4n45:B	(291,298) (290,304)	-0.571	11.000	4n45:B (319, 331)	0.147
1961	211	GGPYAQAA	G2Y3A	GGPYAQAAARALLENT	1g4b:N	(125,132) (125,140)	-0.583	10.250		0.173
2005	212	GDVYTSVA	G2Y3A	TVGDVYTSVAV	2cgg:A	(63,70) (61,71)	-0.589	8.250		0.247
2006	213	GQIYGGLA	G2Y3A	NKLVVDGQIYGGLAQGVGLALS	1dgj:A	(806,813) (800,821)	-0.605	10.750	1dgj:A (840, 844)<br>1dgj:A (869, 873)<br>1dgj:A (874, 888)<br>1dgj:A (897, 904)	0.153
2018	214	GSGYRFIA	G2Y3A	GSGYRFIADKVIEIDR	4pw4:A	(799,806) (799,814)	-0.654	9.375	4pw4:A (826, 828)<br>4pw4:A (834, 850)<br>4pw4:A (855, 867)	0.200
2027	215	GVAYSSGA	G2Y3A	YAYSGVAYSSGALN	2yof:A	(104,111) (100,113)	-0.724	10.750	2yof:A (155, 167)<br>2yof:A (168, 170)	0.144
2045	216	GPYYIDRA	G2Y3A	WGPYYIDRAKAVLD	3s99:A	(250,257) (249,262)	-0.725	8.500		0.227
2046	217	GGSYGWQA	G2Y3A	GGSYGWQAAE	1s20:F	(120,127) (120,129)	-0.726	10.000	1s20:F (304, 317)	0.172
2056	218	GKGYGQTA	G2Y3A	GKGYGQTASGRSRGAVAAAEK	3iib:A	(167,174) (167,187)	-0.750	8.750	3iib:A (419, 423)	0.216
2057	219	GQRYPVIA	G2Y3A	GWGQRYPVIAQMVQYH	4c1r:C	(49,56) (47,62)	-0.766	17.125	4c1r:C (73, 84)	-0.095
2061	220	GQPYQEGA	G2Y3A	LGQPYQEGAG	4q6w:B	(264,271) (263,272)	-0.782	9.000		0.205
2065	221	GKGYSLGA	G2Y3A	TSDVGKGYSLGAELAFKYFNEKG	3eaf:A	(62,69) (58,80)	-0.841	11.250	3eaf:A (273, 276)<br>3eaf:A (279, 285)<br>3eaf:A (323, 327)<br>3eaf:A (328, 346)	0.117
2066	222	GPCYSTKA	G2Y3A	GIGPCYSTKAS	1p9b:A	(146,153) (144,154)	-1.183	9.375		0.160
2067	223	GHPYYSSA	G2Y3A	GVEEEAKIKAPILEQYEHQGHPYYSSAR	3u9r:B	(517,524) (498,525)	-1.186	11.000		0.100
2068	224	GPTYSSKA	G2Y3A	GIGPTYSSKAA	1j4b:A	(168,175) (166,176)	-1.268	8.875		0.172
2079	225	GPVYSSKA	G2Y3A	GIGPVYSSKAA	2v40:A	(167,174) (165,175)	-1.285	9.125	2v40:A (187, 206)	0.162

Supplementary Figure 9. 225 unique helical sequences obtained from the TP-DB for the pattern GY***A given the query of [G 2 Y 3 A].** All the matched patterns are colored in blue, and the residues in bold mean the anchoring residues of matched pattern assumed to mediate the Sgo1-PP2A interaction. The result page is available online at https://dyn.life.nthu.edu.tw/design/result?JobID=612acb53t

#	U#	Matched Sequence (MS)	Matched Pattern	Full Helix (FH)	PDB ID: Chain	Positions in PDB (MS), (FH) & File Download	Helical Propensity	Contact	Interacting Partners	Helicity%
1	1	ANKLKQAVGDIY	A3K3G2Y	DANKLKQAVGDIYN	4nzt:M	(366,377) (365,378)	0.507	8.667	4nzt:M (406, 420)	0.315
2	2	ANSQKGTAGYIY	A3K3G2Y	SWDDWDYAAKVTLANSQKGTAGYIYRFLHD<br>VSE	1qmd:B	(226,237) (213,245)	-0.225	10.000		0.210
14	3	ANSQKGTSGYIY	A3K3G2Y	SWDEWDYAAKVALANSQKGTSGYIYRFLHD<br>VSD	1kho:A	(226,237) (213,245)	-0.720	10.000		0.172

Supplementary Figure 10. Results obtained from the TP-DB for pattern A*K***G**Y with query [A 3 K 3 G 2 Y].** All the matched patterns are colored in blue, and the residues in bold mean the anchoring residues of matched pattern assumed to mediate the Sgo1-PP2A interaction. The result page is available online at https://dyn.life.nthu.edu.tw/design/result?JobID=612acd1di

#	U#	Matched Sequence (MS)	Matched Pattern	Full Helix (FH)	PDB ID: Chain	Positions in PDB (MS), (FH) & File Download	Helical Propensity↕	Contact↕	Interacting Partners	Helicity%↕
1	1	KAAEGLKYEDFA	K3G2Y3A	GESLKKAAEGLKYEDFAK	1exe:A	(87,98) (82,99)	1.264	10.000		0.323
2	2	KKEIGRSYRFIA	K3G2Y3A	KKEIGRSYRFIARN	1ais:B	(1183,1194) (1183,1196)	0.230	9.167		0.275

Supplementary Figure 11. Results obtained from the TP-DB for pattern K*G**Y***A with query [K 3 G 2 Y 3 A]**. All the matched patterns are colored in blue, and the residues in bold mean the anchoring residues of matched pattern assumed to mediate the Sgo1-PP2A interaction. The result page is available online at https://dyn.life.nthu.edu.tw/design/result?JobID=612acd77l

#	U#	Matched Sequence (MS)	Matched Pattern	Full Helix (FH)	PDB ID: Chain	Positions in PDB (MS), (FH) & File Download	Helical Propensity↕	Contact↕	Interacting Partners	Helicity%↕

Supplementary Figure 12. Results obtained from the TP-DB for pattern A*K***G**Y***A with query [A 3 K 3 G 2 Y 3 A].** No peptide found. The result page is available online at https://dyn.life.nthu.edu.tw/design/result?JobID=612ace02p

#	U#	Matched Sequence (MS)	Matched Pattern	Full Helix (FH)	PDB ID: Chain	Positions in PDB (MS), (FH) & File Download	Helical Propensity↕	Contact↕	Interacting Partners	Helicity%↕
1	1	ADEKKFWGKYL	A3K2G2A	EPDTLCAEFKADEKKFWGKYLYEVARRH	4luf:A	(128,138) (118,145)	0.835	8.182	4luf:A (172, 222)	0.358
21	2	AYAKKVEGDMY	A3K2G2A	DRRMENLVAYAKKVEGDMYESAN	2lxt:A	(630,640) (622,644)	0.703	8.727	2lxt:B (846, 856)	0.328
31	3	ARFRKDRGALY	A3K2G2A	DLAAEARFRKDRGALYREAE	1wiy:B	(181,191) (176,195)	0.646	9.364	1wiy:B (207, 238)	0.300

Supplementary Figure 13. Results obtained from the TP-DB for pattern A/***K**/***G**/***Y**/***A with query [A 2,3 K 2,3 G 2,3 Y 2,3 A].** All the matched patterns are colored in blue, and the residues in bold mean the anchoring residues of matched pattern assumed to mediate the Sgo1-PP2A interaction. The result page is available online at https://dyn.life.nthu.edu.tw/design/result?JobID=612ace6bu

We selected the helical PP2A-like peptides found from queries [A 3 K 3 G 2 Y] (**Supplementary Figure 10**), [K 3 G 2 Y 3 A] (**Supplementary Figure 11**), [A 2,3 K 2,3 G 2,3 Y 2,3 A] (**Supplementary Figure 13**), as well as the wide-type PP2A helix as control, for further investigations through all-atom MD-based binding free energy change (ΔG) evaluation by MM/PBSA (see Supporting Methods). The results (**Supplementary Table 2**) showed that seven of the newly found helixes have a binding ΔΔG < 0.0 kcal/mol, indicating their stronger bonding with Sgo1 than the wide-type PP2A. Among them, the number one peptide "**A**YA**K**KVE**G**DM**Y**", having a binding affinity ~10 kcal/mol stronger than the wild-type peptide, can be a promising peptide subject to further experimental validation (e.g. by calorimetry (ITC) assays and/or single molecule experiments). It can be noted that helical propensity does provide a suggestion of helicity percentage to PP2A mutants. Taking the peptides in **Supplementary Table 2** for example, 500 ns simulations showed that the sequence **A**RFR**K**DR**G**AL**Y**REA (1wiy:B) had a higher helicity than

ANSQ**K**GTS**GY**I**Y** (1kho:A) (**Supplementary Figure 14**). Both peptides were derived from the wide type PP2A, **K**TIH**G**LI**Y**, where the substituted consecutive residues "TIH" and "LI", having a helical propensity of -0.139 and 0.153 respectively, were replaced by "DR" and **"**AL**",** having a log(HP_{NA}) of 0.016 and 0.452**,** for 1wiy:B and replaced by "GTS" and **"**YI**",** having a log(HP_{NA}) value of -0.735 and 0.1**,** for 1kho:A. Compared with PP2A (**K**TIH**G**LI**Y**), 1wiy:B that has a higher log(HP_{NA}) of the helical substituents showed a higher helicity percentage than 1kho:A that has a lower log(HP_{NA}) of helical substituents, demonstrated by MD simulations (**Supplementary Figure 14**). Peptide design is an elaborated miniature of protein design, and we expect that herein proposed methodologies can be applied for the design of therapeutic α-helical peptides for other medicinal purposes.

Supplementary Table 2. Seven of the newly found helixes, with binding ΔΔG less than 0.0 kcal/mol, have the potentials of binding stronger to SGO1's helix than the control

Candidate Helix (Matched Pattern)	Helix Source, PDB ID: Chain	Helical Propensity Score	Contact Number	Binding ΔΔG (kcal/mol)	In which Figure above* (U#)
**A**Y**A**K**K**VE**G**DM**Y** (**A 2,3 K 2,3 G 2,3 Y**)	2lxt:A	0.703	8.545	-10.11 ± 0.43	Supplementary Figure 13 (2')
**K**KEI**G**RS**Y**RFI**A** (**K 3 G 2 Y 3 A**)	1ais:B	0.230	9.167	-9.20 ± 0.32	Supplementary Figure 11 (2)
**K**AAE**G**LK**Y**EDF**A** (**K 3 G 2 Y 3 A**)	1exe:A	1.264	10.000	-8.04 ± 0.29	Supplementary Figure 11 (1)
**A**Y**A**K**K**VE**G**DM**Y**ES**A** (**A 2,3 K 2,3 G 2,3 Y 2,3 A**)	2lxt:A	0.703	8.727	-6.64 ± 0.45	Supplementary Figure 13 (2)
**A**NSQ**K**GTA**G**YI**Y** (**A 3 K 3 G 2 Y**)	1qmd:B	-0.225	10.000	-5.63 ± 0.33	Supplementary Figure 10 (2)
**A**RFR**K**DR**G**AL**Y**RE**A** (**A 2,3 K 2,3 G 2,3 Y 2,3 A**)	1wiy:B	0.646	9.364	-1.96 ± 0.46	Supplementary Figure 13 (3)
**A**RFR**K**DR**G**AL**Y** (**A 2,3 K 2,3 G 2,3 Y**)	1wiy:B	0.957	8.500	-1.14 ± 0.38	Supplementary Figure 13 (3')
**K**TIH**G**LI**Y** **Control, from PP2A**	3fga:B	-0.200	9.250	0.00	
**A**NSQ**K**GTS**GY**I**Y** (**A 3 K 3 G 2 Y**)	1kho:A	-0.720	10.000	1.38 ± 0.39	Supplementary Figure 10 (3)
**A**DEK**K**FW**G**KY**L**Y (**A 2,3 K 2,3 G 2,3 Y**)	4luf:A	0.957	8.500	4.31 ± 0.29	Supplementary Figure 13 (1')
**A**DEK**K**FW**G**KY**L**YEV**A** (**A 2,3 K 2,3 G 2,3 Y 2,3 A**)	4luf:A	0.835	8.182	4.66 ± 0.86	Supplementary Figure 13 (1)
**A**NKL**K**QAV**G**DI**Y** (**A 3 K 3 G 2 Y**)	4nzt:M	0.507	8.667	5.11 ± 0.32	Supplementary Figure 10 (1)

*The earlier Tables that listed the matched sequences (in blue) are denoted at the rightmost column. The residues in bold mean the anchoring residues of matched pattern assumed to mediate the Sgo1-PP2A interaction, and the number in the parenthesis is the serial number for the unique (U#) sequence in the corresponding Table of interest. A prime following a U#, such as 2', indicates that the sequence in that row of this table is a subsequence of the original sequence presented in the earlier Tables.

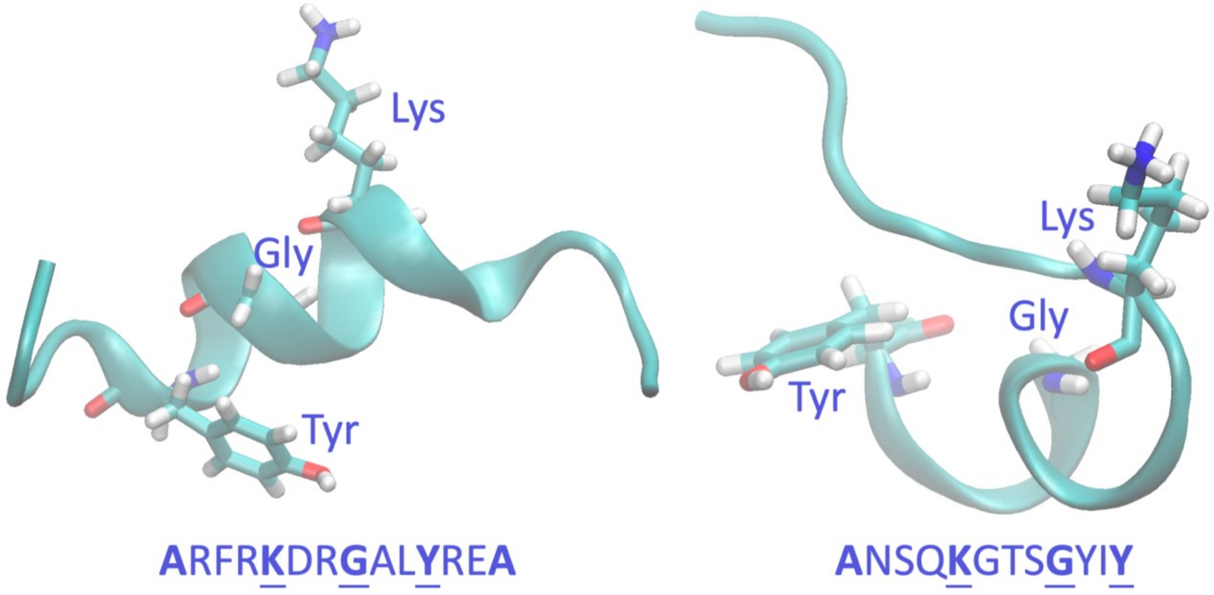

Supplementary Figure 14. Helicity of two TP-DB-suggested PP2A mutants. In **Supplementary** Table 2, **A**RFR**K**DR**G**AL**Y**RE**A** (1wiy:B) are found by 500 ns simulations to have a higher helicity% than **A**NSQ**K**GTS**G**YI**Y** (1kho:A) where the pattern-matched substituent residues in 1wiy:B have a higher log(HP_{NA}) than corresponding ones in 1kho:A. The bold-faced letters are the anchoring residues in the patterns while Lys, Gly and Tyr residues shown as licorice in the figures are underlined.

Supplementary Methods

Production of recombinant Helicobacter pylori (H. pylori) neutrophil-activating protein (HP-NAP) and maltose-binding protein (MBP)

Recombinant *H. pylori* neutrophil-activating protein (HP-NAP) was expressed in *E. coli* BL21(DE3) cells harboring the expression plasmid pET42a-NAP and purified by either two consecutive gel-filtration chromatography as previously described[12] or a small-scale DEAE Sephadex negative mode batch chromatography as previously described[13]. Maltose-binding protein (MBP) was prepared the same as the procedure for production of MBP fused with the polypeptide containing residues Arg77 to Glu116 of HP-NAP as described below except that *E. coli* BL21(DE3) cells harboring the pMALc2g expression vector was used for expression.

Cloning of HP-NAP$_{R77\text{-}E116}$ into a MBP fusion protein expression vector

The plasmid DNA pET42a-NAP encoding a *napA* gene [GenBank:AE000543.1, Gene: HP0243] from *H. pylori* strain 26695 was prepared as previously described[12]. The DNA fragment coding for polypeptide containing residues Arg77 to Glu116 of HP-NAP (HP-NAP$_{R77\text{-}E116}$), which contains the D-Y-K-x-x-[DE] motif, was amplified by PCR from the plasmid pET42a-NAP using the forward and reverse primers containing BamHI and HindIII site, respectively. The forward primer is 5'-ATAAGGATCCCGTGTTAAAGAAGAAACTAAAAC-3' and the reversed primer is 5'-TTAATAAGCTTTAATTCTTTTTCAGCGGTGTTAGAG-3'. The PCR reaction was carried out with 10 ng plasmid DNA pET42a-NAP as a template and KAPA HiFi PCR Kit (Kapa Biosystems, Inc.) in a Mastercycler Gradient 5331 (Eppendorf, Germany). An initial denaturing phase of 95 °C for 5 min was followed by 39 cycles of 98 °C for 20 sec, 67 °C for 15 sec, and 72 °C for 15 sec. A final elongation phase of 72 °C for 2 min was also included. The amplified DNA fragments encoding HP-NAP$_{R77\text{-}E116}$ were then cloned into pJET1.2/blunt vectors using the CloneJET PCR Cloning Kit (Thermo Fisher Scientific Inc.). The resulting plasmid was designated as pJET1.2/blunt-HP-NAP$_{R77\text{-}E116}$. The insert was sequenced to confirm the correct DNA sequence. The correct insert was digested from pJET1.2/blunt-HP-NAP$_{R77\text{-}E116}$ with BamHI and HindIII and then cloned into the pMALc2g expression vector[14]. The resulting plasmid was designated as pMALc2g-HP-NAP$_{R77\text{-}E116}$.

Production of MBP-tagged HP-NAP$_{R77-E116}$

E. coli BL21(DE3) cells harboring pMALc2g-HP-NAP$_{R77-E116}$ were streaked on a lysogeny broth (LB) agar plate containing 100 µg/ml ampicillin and incubated at 37 °C for 16 hr. A single colony was picked and inoculated into 4 ml of LB containing 100 µg/ml ampicillin and the culture was incubated at 37 °C with shaking at 170 rpm for 16 hr. A volume of 2 ml of the overnight culture was inoculated into 200 ml LB containing 100 µg/ml ampicillin and the inoculated culture was incubated at 37 °C with shaking at 170 rpm for 2 hr until the OD_{600} reached 0.5. The expression of MBP-tagged HP-NA$_{PR77-E116}$ was induced by the addition of isopropyl β-D-1-thiogalactopyranoside (IPTG) to a final concentration of 0.3 mM and the culture was incubated at 37 °C with shaking at 180 rpm for 3 h until the OD_{600} reached 1.7. Then, the cells were centrifuged at 6,000 x g at 4 °C for 15 minutes to remove the supernatant and the cell pellets were stored at -70 °C.

The cell pellet from a 200 ml culture of *E. coli* expressing recombinant MBP-tagged HP-NAP$_{R77-E116}$ were re-suspended in 20 ml of ice-cold buffer containing 20 mM Tris-HCl, pH 7.4, 200 mM NaCl, 1 mM ethylenediaminetetraacetic acid (EDTA), and 1 mM dithiothreitol (DTT), plus 0.1% (v/v) protease inhibitor mixture (PI mix). The PI mix contained 0.13 M phenylmethylsulfonyl fluoride (PMSF), 0.03 M N-alpha-tosyl-L-lysyl-chloromethyl ketone (TLCK), and 0.03 M N-tosyl-L-phenylalanyl-chloromethyl ketone (TPCK). The bacterial suspensions were disrupted by Emulsiflex C3 high-pressure homogenizer (Avestin) operated at a range of 15,000-20,000 psi for 7 times at 4 °C. The lysates were centrifuged at 30,000 x g at 4 °C for 1 hr to separate insoluble and soluble proteins by using a Hitachi himac CP80WX ultracentrifuge (Hitachi Koki Co. Ltd., Tokyo, Japan). Then, 5 mL supernatant containing the soluble proteins were loaded onto a 1-ml MBPTrap HP column (GE Healthcare Bio-Sciences), which was pre-equilibrated with 20 mM Tris-HCl, pH 7.4, 200 mM NaCl, 1 mM EDTA, and 1 mM DTT, at a flow rate of 0.5 ml/min at 4 °C by ÄKTA Purifier. The column was eluted with 20 mM Tris-HCl, pH 7.4, 200 mM NaCl, 1 mM EDTA, 1 mM DTT, and 10 mM maltose at a flow rate of 1 ml/min at 4 °C by ÄKTA Purifier. The flow-through and elution fractions were analyzed by SDS-PAGE on a 12% gel. The elution fractions containing recombinant MBP-tagged HP-NAP$_{R77-E116}$ were collected and concentrated to a concentration higher than 1 mg/ml.

Recombinant HP-NAP-based enzyme linked immunosorbent assay (ELISA)

Nunc MaxiSorp ninety-six-well enzyme linked immunosorbent assay (ELISA) plates (Nunc, Rochester, NY, USA) were coated with 0.3 μg of recombinant HP-NAP in 100 μl of carbonate-bicarbonate buffer, pH 9.6, for each well at room temperature for 16 hr. Each well was washed three times with 300 μl of phosphate buffered saline (PBS), pH 7.4, containing 20 mM Na_2HPO_4, 1.47 mM KH_2PO_4, 137 mM NaCl, and 2.7 mM KCl, with the addition of 0.1% tween-20 (PBS-T) for 10 min each time. The wells were blocked with 250 μl PBS with 1% bovine serum albumin (BSA) for 2 hr and then washed three times with 300 μl of PBS-T buffer for 10 min each time. The anti-FLAG M2 antibody (Sigma-Aldrich, Cat# F-3165) and its corresponding mouse IgG antibody (Sigma-Aldrich, Cat# I5381) at a concentration of 600 ng/ml and the hybridoma culture supernatant containing mouse monoclonal antibody MAb 16F4[15] against HP-NAP at a dilution of 1:5000 in 100 μl of PBS-T buffer containing 1% BSA were added into each well. The plate was incubated at room temperature for 1 hr and then the wells were washed three times with 300 μl of PBS-T buffer for 10 min each time. The horseradish peroxidase-conjugated goat anti-mouse secondary antibody (Jackson ImmunoResearch, Cat#115-035-003) at a dilution of 1:10000 in 100 μl of PBS-T buffer containing 1% BSA was loaded into each well. The plate was incubated at room temperature for 1 hr and then the wells were washed three times with 300 μl of PBS-T buffer for 10 min each time. The color was developed using 3,3',5,5'-tetramethylbenzidine (TMB) peroxidase substrate (Thermo Scientific). The reaction was terminated by the addition of 2 N H_2SO_4, and the absorbance at 450 nm was measured by an iMark microplate absorbance reader (Bio-rad, Hercules, CA).

Western blot analysis

Western blotting was performed essentially the same as previously described[16]. The membrane was probed with either anti-FLAG M2 antibody (Sigma-Aldrich, Cat# F-3165) at a concentration of 1 μg/ml or the hybridoma culture supernatant containing mouse monoclonal antibody MAb 16F4[15] against HP-NAP at a dilution of 1:2000.

Secondary structure determined by circular dichroism (CD) spectra

Circular Dichroism (CD) spectra[17] measurement over the wavelength 190 to 260 nm was conducted for the peptides in Table 3 to understand their helicity percentage in isolation. All the peptides of 60 μM were prepared in a 20 mM phosphate buffer (pH 7.4) containing 25 mM DPC loaded to a 1 mm quartz cuvette for the detection of CD spectrometer (Model MOS500, BioLogic, Seyssinet-Pariset, France). The spectra of CM15 and a cell penetrating peptide, Tat, in the same concentration were also measured as the helical control and the random coil control, respectively. The far-UV CD spectra from 190 to 260 nm were recorded at 37°C in the interval of 0.5 nm. Data averages were taken from 3 scans by baseline subtraction of buffer blank. The mean residue ellipticity (MRE) in deg cm^2 $dmol^{-1}$ was obtained as

$$[\theta] \text{ molar ellipticity (deg cm}^2\text{ dmol}^{-1}) = \theta_{obs} / (l \times C \times n_{pb}) \quad (1)$$

where 'θ_{obs}' is average ellipticity from CD spectra in millidegrees, 'C' is peptide concentration in molarity, 'l' is path length of cuvette in centimeter, and 'n_{pb}' is the number of peptide bonds (peptide length -1).

Selection of the helical AMPs in isolation from the PDB (Isolated Peptide Set)

On the foundation of our previously published iGNM 2.0 server[18] to survey the PDB files containing peptides (<20 amino acids) with low contact (<7.5 amino acids in average using a 7.3Å $C\alpha$-$C\alpha$ cutoff[18] of which the helical stretches can also be found in TP-DB, we notice that almost all the isolated (low-contact bearing) peptides, having a high portion of helical stretches, are antimicrobial peptides, many of which are solved by NMR. We therefore searched the PDB using its Advanced Search function, setting the Description/Structure Title attribute to be "Antimicrobial Peptide" and experimental method attribute to be "Solution NMR" or "Solid-State NMR". NMR instead of x-ray crystallography was chosen as a preferred method because we want to avoid the possibility of secondary structures in some isolated peptides being stabilized by crystal contacts. According to the procedure, we obtained 80 peptides with unique PDB IDs, where 54 of them contain helical stretches. Their PDB IDs are 2l24, 6vla, 2l3i, 5ykk, 2n8d, 2gdl, 2hfr, 2lg4, 5ki0, 1xkm, 2kus, 2mjq, 2l1q, 2mlv, 2mlu, 6j9p, 2mxh, 2l5r, 2m6a, 2n1c, 6fs4, 6fs5, 2knj, 2rlh, 2rlg,

2amn, 1mm0, 2n1s, 2pco, 5ykl, 6twg, 5x3l, 6wpb, 6wpd, 6wpo, 6t33, 2dd6, 2g9p, 1og7, 1ohn, 2igr, 2n9r, 1ry3, 1t51, 1t55, 1t52, 2k98, 1d6x, 2g9l, 2l2r, 6ct4, 1ot0, 1p0g, 6n68.

From the literature survey for each of these published AMPs, we further removed those structurally solved in the presence of nanoparticles and those whose helical portion can be stabilized by disulfide bonds, leaving a set of 37 peptides whose helicity percentage is listed in **Supplementary Table 3**. Here, helicity % is defined as the number of amino acids in their helical forms over the length of the entire sequence. We coin this set in **Supplementary Table 3** as the "isolated peptide" set.

Selection of TP-DB peptides with specific ranges of helical propensity and average residue contacts (TP-DB peptide set) for MD simulations that reveal their helicity percentage

As shown in **Supplementary Figure 2**, the distributions of the helical propensity, log (HP_{NA}), and the average contact number for all the TP-DB helices reveal an average helical propensity and contact of 0.38 and 8.87, respectively, with the most populated bins being 0.2 to 0.4 for the helical propensity and 8.00 to 8.25 for the average contact. With that, we took 15 TP-DB peptides having helical propensity of 0.2 to 0.4 with a range of average residue contact from 7 to 11; in parallel, we also took another 8 peptides having a contact of approximately 8.1 with a range of helical propensity from -0.5 to 2.0. We term the collection of these 23 peptides as the TP-DB peptide set (listed in **Supplementary Table 4**). We also noticed in relevant MD simulations that the presence of proline tends to break the helices due to its special backbone/side-chain connectivity, which aggravates particularly for peptides in isolation when the 3D contact no longer supports the integral local environment for proline residues in the protein helices. Therefore, we do not include any peptides containing a proline in our TP-DB set. According to our statistics, proline-contained TP-DB peptides comprise less than ¼ of the entire database.

Least-squares fitting to find helicity% as functions of helical propensity, concentration and tertiary contact

Let $\vec{x_i} = [1, x_1^i, x_2^i]$ where x_1 is the helical propensity and x_2 is either concentration or tertiary contact, where i is the index for individual peptides. The helicity percentage y_i can be expressed

as a dot product of the independent variable $\vec{x_i}$ and their parameters $\vec{\beta} = [\beta_0, \beta_1, \beta_2]$, where β_1 is the coefficient of x_1 and β_2 is the coefficient of x_2. We can write

$$y_i \approx \sum_{j=0}^{2} \beta_j \times x_j^{\,i} = \vec{\beta} \cdot \vec{x_i} \qquad (2)$$

such that the experimentally observed or MD-determined helicity% on the left of the equal sign can be approximated by the dot product on the right. Here, i =1 to 37 or 41 for the AMP case and i =1 to 23 for isolated TP-DB peptides and therefore the n in the **equation (2)** below is 37, 41 or 23.

In the least-squares fitting[19], the optimum parameters $\hat{\vec{\beta}}$ can be obtained from the minimized sum of mean squared loss $\sum_{i=1}^{n} (\vec{\beta} \cdot \vec{x_i} - y_i)^2$ such that

$$\hat{\vec{\beta}} = arg_{\vec{\beta}} min \sum_{i=1}^{n} (\vec{\beta} \cdot \vec{x_i} - y_i)^2 = (\mathbf{X}^T\mathbf{X})^{-1}\mathbf{X}^T\mathbf{Y} \qquad (3)$$

where **X** is a $n\times3$ matrix constituted with the helical propensity and concentration (or tertiary contact) of n peptides and **Y** is a $n\times1$ matrix comprising n data points of experimentally observed or MD-determined helicity%. The matrix calculations were carried out using the programming language MATLAB.

Finding PP2A-like peptides

Knowing that the K374, G378, and Y381 of PP2A plays important roles in PP2A's favourable interactions with Sgo1 (see **Supplementary Fig. S5**), we set out to search for a peptide with a similar amino acid pattern and which may interact better than with Sgo1 and thereby out compete/displace PP2A and bind to Sgo1. Therefore, we searched the developed TP-DB for the following queries: **[K 3 G 2 Y], [A 3 K 3 G], [G 2 Y 3 A], [A 3 K 3 G 2 Y], [K 3 G 2 Y 3 A], [A 3 K 3 G 2 Y 3 A], and [A 2,3 K 2,3 G 2,3 Y 2,3 A].** The resulting peptides from each query are ranked based on their helical propensity score and contact number.

Minimization and MD simulations of Martini Coarse-Grained (CG) models in vacuum and assessing the stability of complexes

To search for PP2A-like helical peptides to block the interaction between Sgo1 and PP2A, 69 sequences (https://dyn.life.nthu.edu.tw/design/result?JobID=602bae01v) that matched the pattern "K***G**Y" in PP2A helix (see **Supplementary Fig. S5**; the three anchoring residues of PP2A helix **K**$_{374}$**T**$_{375}$**I**$_{376}$**H**$_{377}$**G**$_{378}$**L**$_{379}$**I**$_{380}$**Y**$_{381}$ that interacts with Sgo1 are K374, G378 and Y381) were chosen for affinity assessment. Methodologically, it was equivalent to computationally modify (mutate) the five spacing residues T375, I376, H377, L379 and I380 in PP2A helix "KTIHGLIY" into corresponding residues in every of these 69 sequences. In other words, we only replaced the alpha-helical segment "K$_{374}$TIHGLIY$_{381}$" in PP2A [PDB:3FGA; chain B] by every of the 69 helical stretches that matched the pattern "K***G**Y" and the segment was shown to interact with the helix "V$_{75}$KEAQDIILQLRKECYYL$_{92}$" in Sgo1 [PDB:3FGA; chain D]. Subsequently, all 69 complexes comprising the Sgo1 helix and mutated PP2A helix were converted into coarse-grained (CG) models by the web service CHARMM-GUI[20]. The 69 CG models of complexes containing PP2A mutants and the CG model for the wide-type complex were energy-minimized and then briefly equilibrated for 1 ps at 20 fs time step by MD simulations where the backbone nodes were restrained at whole time (spring constant = 2.5 kcal/mol/Å^2). The MD simulations were run with GROMACS package[21] using MARTINI forcefield[22,23]. The pressure and temperature were maintained at 1 bar and 310K, respectively. The cutoff distances for both Coulomb and Van der Waal were set to be 12Å. The potential energies at the final step were recorded to represent the binding stability between Sgo1 and mutated (or wild-type) PP2A.

Besides the top-ranked peptides by Martini forcefield from the aforementioned 69 complexes, we also searched potential helical binders from TP-DB using at least 4 anchoring residues in the search pattern (**Supplementary Figures 10** to **13**). We carried out all-atom molecular dynamics simulations for these peptides to assess their interactions with the targeted helix of Sgo1 using MM/PBSA[24,25] (see below). The top 11 identified helices (**Supplementary Table 2**), searched from the queries [A 3 K 3 G 2 Y], [K 3 G 2 Y 3 A] and [A 2,3 K 2,3 G 2,3 Y 2,3 A], were first superimposed onto the template helix of the PP2A [PDB: 3FGA; chain B; residue 372 to 383] with their common residues K374, G378, and Y381 (as shown in **Supplementary Figure 5**), before the simulations.

We carried out the all-atom molecular dynamics simulations by AMBER16[26,27] package, using ff14SB forcefield[28] for protein and ionsjc_tip3p for ions[29] in explicit solvent. Each molecular system to be simulated was placed in a periodic box and solvated with TIP3P water. All the input files for MD simulations were prepared using tLeap[26] from AmberTools16.

Energy minimization was performed in three stages - first, with weak harmonic positional restraints (spring constant = 0.5 kcal/mol/Å^2) on all atoms except for the water/solvent atoms; second, with weak harmonic restraints on the CA atoms of amino acids; lastly, without restraints. Each of the first two stages of energy minimization is composed of 5,000 steps, with the first 2500 steps (for each energy minimization stage) carried out using steepest descent algorithm and the remaining 2,500 steps carried out using conjugate gradient algorithm.

Following the energy minimization, each simulation system was slowly heated to 310K while applying weak harmonic positional restraints on the CA atoms. Each system was then equilibrated at the temperature reached without any positional restraints prior to production MD simulations. Each production MD simulation ran for 500 ns at 2 fs time step. Non-bonded interactions were evaluated up to a cut-off distance of 10 Å where it was switched off with a cubic spline switch function. Particle Mesh Ewald method[30] was used to calculate full electrostatic interactions energies. All temperature regulations were done using Langevin thermostat (with a collision frequency, γ, of 2 ps^{-1}) and all pressure control was done with Berendsen barostat[31]. Binding free energy change (binding ΔG) was calculated from the MD simulation trajectories using AMBER16's implementation of Molecular Mechanics/Poisson-Boltzmann Surface Area (MM/PBSA)[24,25].

Supplementary Table 3. **The helical propensities and observed helicity percentage of the 37 isolated NMR-resolved peptides**

PDB	Full sequence*	Helical propensity ¶	Helicity percentage	Concentration (mM) §	Literature
6FS5	KTKLTEEEKNRLNFLKKISQRYQK FALPQYLKTVYQHQK	2.137	**34** /39 (87.18%)	0.95	32
6FS4	TKLTEEEKNRLNFLKKISQRYQKF ALPQYLK	1.775	**18** /31 (58.06%)	1.2	32

2DD6	ALWKTLLKKVLKA	1.695	**7** /13 (53.85%)	2.2	33
2IGR	KWKVFKKIEKKWKVFKKIEKAGP KWKVFKKIEK	1.448	**31** /33 (93.94%)	3	34
2PCO	SMWSGMWRRKLKKLRNALKKKL KGEK	1.44	**18** /26 (69.23%)	2	35
1OT0	AKKVFKRLEKLFSKIQNWK	1.426	**16** /19 (84.21%)	1	36
2RLH	ALYKKFKKKLLKSLKRLG	1.339	**16** /18 (88.89%)	1.5	37
2RLG	ALYKKFKKKLLKSLKRLG	1.339	**15** /18 (83.33%)	1.5	37
6J9P	RRLIRLILRLLR	1.156	**11** /12 (91.67%)	1.5	38
1P0G	AKKVFKRLEKLFSKIQNDK	1.127	**13** /19 (68.42%)	1	36
2L3I	GIRCPKSWKCKAFKQRVLKRLLA MLRQHAF	0.951	**17** /30 (56.67%)	1	39
6CT4	PMKKLKLALRLAAKIAPVW	0.834	**13** /19 (68.42%)	1	40
6WPD	GILDAIKAIAKAAG	0.773	**13** /14 (92.86%)	1	41
2N9R	PKILNKILGKILRLAAAFK	0.587	**17** /19 (89.47%)	1	42
2N8D	WDPYFAGVKKLTKAILAVRA	0.56	**12** /20 (60%)	0.0089	43
2AMN	RVKRVWPLVIRTVIAGYNLYRAIK KK	0.167	**17** /26 (65.38%)	4	44
6TWG	FLPKILRKIVRAL	0.149	**12** /13 (92.31%)	0.86	45
6VLA	LMGLFNRIIRKVVKLFN	0.076	**16** /17 (94.12%)	1.28	46
2G9P	GLFGKLIKKFGRKAISYAVKKARG KH	-0.054	**22** /26 (84.62%)	1.7	47
2L24	IFGAIAGFIKNIW	-0.149	**11** /13 (84.6%)	2	48
1T51	ILGKIWEGIKSLF	-0.152	**11** /13 (84.62%)	1	49
2L5R	GLKEIFKAGLGSLVKGIAAHVAS	-0.319	**22** /23 (95.65%)	1.5	50
1T55	ILGKIWKPIKKLF	-0.343	**6** /13 (46.15%)	1	49
2HFR	KRFWPLVPVAINTVAAGINLYKAI RRK	-0.35	**11** /27 (40.74%)	4	51
6N68	ILGTILGLLKGL	-0.453	**6** /12 (50%)	1	52

1D6X	VRRFPWWWPFLRR	-0.57	**5** /13 (38.46%)	3	53
2MLV	MKTILRFVAGYDIASHKKKTGGYP WERGKA	-0.766	**15** /30 (50%)	1	54
2MLU	MKTILRFVAGYDIASHKKKTGGYP WERGKA	-0.766	**16** /30 (53.33%)	1	54
2K98	GIGKFLKKAKKGIGAVLKVLTTGL	-0.769	**22** /24 (91.67%)	0.5	55
2GDL	LVQRGRFGRFLRKIRRFRPKVTITI QGSARF	-1.222	**17** /31 (54.84%)	3	56
2N1C	FEDLPNFGHIQVKVFNHGEHIHH	-1.461	**15** /23 (65.22%)	3.5	57
2MXH	VARGWKRKCPLFGKGG	-1.668	**12** /16 (75%)	0.5	N/A ¥
5X3L	GLGSVFGRLARILGRVIPKV	-2.029	**9** /20 (45%)	1.5	58
1OG7	KYYGNGVHCGKHSCTVDWGTAI GNIGNNAAANWATGGNAGWNK	-2.501	**16** /43 (37.21%)	1	59
1OHN	KYYGNGVHCGKHSCTVDWGTAI GNIGNNAAANWATGGNAGWNK	-2.501	**19** /43 (44.19%)	1	59
5KI0	RAIGGGLSSVGGGSSTIKY	-2.957	**10** /19 (52.63%)	3.4	60
1RY3	MNSVKELNVKEMKQLHGGVNYG NGVSCSKTKCSVNWGQAFQERYT AGINSFVSGVASGAGSIGR	-3.94	**32** / 64 (50%)	1	61

Correlation between helical propensity and helicity percentage	0.50

All the isolated peptides were found to be structurally solved in the presence of micelles or TFE. Here, helicity percentage is defined as the number of amino acids in helical stretches (the number shown in bold face indicate the total number of residues underlined) divided by the length of the full sequence containing these stretches.

* : The underlined stretches are the helical parts of peptides stored in the TP-DB.

¶ : The helical propensities are calculated based on the entire sequence, instead of just the helical parts, of the peptides, summing the log (HP_{NA}) values (see Table 1) for all the amino acids in a peptide.

§ : The concentrations of AMPs used in NMR-based structure determination.

¥ : No relevant publication.

Supplementary Table 4. The MD-sampled helicity percentage for the 23 TP-DB-stored peptides in a range of helical percentage and average residue contact

TP-DB Seq. Identifier	Seq	Avg. Res Contact	Helical Propensity	Max_sum4	Helicity% (50-100ns Avg.)
3da9_B_1_276_288	**LKKW**IQKVIDQFG	7.000	0.339	0.615	0.601
3rmg_B_1_31_44	**ALLD**SVITHIRKRN	7.000	0.256	0.578	0.647
3tlx_D_1_195_207	V**LKKR**LTVFKSET	7.846	0.310	0.43	0.533

4buc_B_1_433_445	KRGEHFR**EIFK**RH	7.856	0.277	0.277	0.673
3los_A_1_147_159	**KEIL**TKIAMTSIT	9.000	0.396	0.468	0.288
1n4s_H_1_21_33	**LRDR**HVRFFQRCL	9.000	0.399	0.256	0.64
1rx0_C_1_79_92	SRLDTSVIF**EALA**T	11.000	0.393	0.949	0
4fl4_J_1_32_44	NSTDLT**LLKR**YVL	11.000	0.376	0.51	0.609
2zzd_K_1_3_16	SS**IREE**VHRHLGTV	8.100	-0.498	0.483	0.498
3e2r_A_1_140_152	NASGRAGMV**QGLL**	8.200	-0.499	0.054	0.006
3oka_B_1_19_31	GGIQS**YLRD**FIAT	8.200	-0.500	0.313	0.017
3i6s_A_1_445_458	**NKKE**GKQVINYVKN	8.200	0.001	0.344	0.027
1x8k_A_1_131_144	DMERYLG**LLEY**SSR	8.000	0.501	0.692	0.188
3ist_A_1_240_253	STQIFKDI**AKDW**LN	8.000	0.501	0.573	0.017
3n0x_A_1_199_211	FTAVGQRLF**DALK**	8.100	0.501	0.498	0.149
2fw2_E_1_190_203	TFTQEVMIQI**KELA**	8.000	1.000	0.767	0.464
3g6w_B_1_146_159	ASTMLK**VLEE**VVKA	8.200	1.000	0.512	0
3p3q_A_1_184_196	GEALI**ALER**ERFA	8.200	1.504	0.737	0.029
1qh8_B_1_396_408	KR**WQKA**MNKMLDA	8.100	1.502	0.757	0.048
1sus_B_1_43_55	HEAMK**ELRE**VTAK	8.200	1.501	0.68	0.647
2p6f_D_1_6_19	KAKKLENLLK**LLQL**	8.100	1.999	0.66	0.551
1t9m_B_1_150_163	DIHAL**RAEA**RRLAE	8.000	1.992	0.839	0.619
**1smq_B_1_93_105**	YHEIWQAYK**RAEA**	8.200	2.009	0.839	0.538
	**Correlation**	**-0.573**	**0.510**	**0.549**	
**1smq_B_1_93_105 Mutants**					
Random_shuffled 1	EKHRIAAYQ**WYEE**		2.009	0.812	0.366
Random_shuffled 2	YE**QAWA**HYKAEII		2.009	0.939	0.705
Random_shuffled 3	AAQYYWERH**AKEE**		2.009	0.812	0
Random_shuffled 4	**AAEQ**AWRYYEKHH		2.009	0.909	0.639
Random_shuffled 5	**WAAR**QYYIHAKEE		2.009	0.869	0.019
	**Correlation**			**0.631**	

Residues underlined in bold are the location of folding cores, having the highest sum of log(HP_{NA}) values (Max_sum4) among all the 4-residue windows in that peptide. All the correlation coefficients in the Table are the correlation between the parameters in the same column as where the coefficient is listed and the corresponding helicity percentage (Helicity%) of the peptides. However, the correlation (in bold) for Avg. Res. Contact takes account only the top 8 peptides (in dark red), while Helical Propensity and Max_sum4 consider all the peptides having an Avg. Res. Contact of 8.0 to 8.2 (the 9th to the 23rd peptide in cyan and violet, respectively). The five mutants of 1smq_B_1_93_105, YHEIWQAYK**RAEA**, are listed at the bottom of the Table, shaded by a lemon green color. Together with the "wild type", the Max_sum4 values for all the 6 peptides, shaded in light gray, are found to correlate with Helicity% with a 0.63 correlation coefficient with the relation Helicity% = 3.70 Max_sum4 - 2.82.

Supplementary Table 5. Validation of regression-model-predicted helicity percentage using newly published NMR-solved isolated peptides

PDB	Full sequence*	Helical propensity ¶	Conc. (mM) §	Observed helicity %	Predicted helicity % ¥
7MMM	IWLTALKFLGKNL GKHLAKQQLAKL	1.968	2.0	**23 /25** (92.00%)	81.19%
7M67	GILDIKNKVSNLFK KIKGEK	-0.306	1.0	**17 /20** (85.00%)	69.41%
Estimated error £					13.4%

* : The underlined stretches are the helical parts of peptides shown in the PDB, and the lengths of stretches are divided by their full length to obtain the observed helicity% shown in bold

¶ : The helical propensities are calculated based on the entire sequence, instead of just the helical parts, of the peptides, summing the log (HP_{NA}) values (see Table 1) for all the amino acids in a peptide.

§ : The concentrations of AMPs used in the NMR-based structure determination.

¥ : As shown in Fig 6, Predicted helicity % = 0.744 - 0.03 concentration (in mM) + 0.065 helical_propensity

£: The root mean square deviation (r.m.s.d.) between experimental and predicted helicity % are calculated as the estimated error, where the difference between experimental and predicted helicity % for each data point is squared and the quantity for all the data points are summed, divided by the number of data points and eventually taking square root.

Supplementary References

1. Emmanuel O Salawu, Therapeutic Peptide Design Database (TP-DB), Zenodo, https://doi.org/10.5281/zenodo.5653287, 2021.
2. Eastman, P. *et al.* OpenMM 7: Rapid development of high performance algorithms for molecular dynamics. *PLoS Comput. Biol.* **13**, e1005659 (2017).
3. Klauda, J. B. *et al.* Update of the CHARMM all-atom additive force field for lipids: validation on six lipid types. *J. Phys. Chem. B* **114**, 7830–7843 (2010).
4. Best, R. B. *et al.* Optimization of the additive CHARMM all-atom protein force field targeting improved sampling of the backbone φ, ψ and side-chain χ(1) and χ(2) dihedral angles. *J. Chem. Theory Comput.* **8**, 3257–3273 (2012).
5. Salic, A., Waters, J. C. & Mitchison, T. J. Vertebrate shugoshin links sister centromere cohesion and kinetochore microtubule stability in mitosis. *Cell* **118**, 567–578 (2004).
6. Wang, L.-H. *et al.* Sgo1 is a potential therapeutic target for hepatocellular carcinoma. *Oncotarget* **6**, 2023–2033 (2015).
7. Liu, H., Rankin, S. & Yu, H. Phosphorylation-enabled binding of SGO1-PP2A to cohesin protects sororin and centromeric cohesion during mitosis. *Nat. Cell Biol.* **15**, 40–49 (2013).
8. Tang, Z. *et al.* PP2A is required for centromeric localization of Sgo1 and proper chromosome segregation. *Dev. Cell* **10**, 575–585 (2006).
9. Marston, A. L. Shugoshins: tension-sensitive pericentromeric adaptors safeguarding chromosome segregation. *Mol. Cell. Biol.* **35**, 634–648 (2015).
10. Xu, Z. *et al.* Structure and function of the PP2A-shugoshin interaction. *Mol. Cell* **35**, 426–441 (2009).
11. Phillips, J. C. *et al.* Scalable molecular dynamics with NAMD. *J. Comput. Chem.* **26**, 1781–

1802 (2005).

12. Wang, C.-A., Liu, Y.-C., Du, S.-Y., Lin, C.-W. & Fu, H.-W. Helicobacter pylori neutrophil-activating protein promotes myeloperoxidase release from human neutrophils. *Biochem. Biophys. Res. Commun.* **377**, 52–56 (2008).
13. Yang, Y.-C. *et al.* High yield purification of Helicobacter pylori neutrophil-activating protein overexpressed in Escherichia coli. *BMC Biotechnol.* **15**, 23 (2015).
14. Walker, I. H., Hsieh, P.-C. & Riggs, P. D. Mutations in maltose-binding protein that alter affinity and solubility properties. *Appl. Microbiol. Biotechnol.* **88**, 187–197 (2010).
15. Iankov, I. D., Haralambieva, I. H. & Galanis, E. Immunogenicity of attenuated measles virus engineered to express Helicobacter pylori neutrophil-activating protein. *Vaccine* **29**, 1710–1720 (2011).
16. Hong, Z.-W., Yang, Y.-C., Pan, T., Tzeng, H.-F. & Fu, H.-W. Differential effects of DEAE negative mode chromatography and gel-filtration chromatography on the charge status of Helicobacter pylori neutrophil-activating protein. *PLoS One* **12**, e0173632 (2017).
17. Greenfield, N. J. Using circular dichroism spectra to estimate protein secondary structure. *Nat. Protoc.* **1**, 2876–2890 (2006).
18. Li, H., Chang, Y.-Y., Yang, L.-W. & Bahar, I. iGNM 2.0: the Gaussian network model database for biomolecular structural dynamics. *Nucleic Acids Res.* **44**, D415–22 (2016).
19. Rencher, A. C. & Christensen, W. F. *Methods of Multivariate Analysis*. (John Wiley & Sons, 2012).
20. Lee, J. *et al.* CHARMM-GUI Input Generator for NAMD, GROMACS, AMBER, OpenMM, and CHARMM/OpenMM Simulations Using the CHARMM36 Additive Force Field. *J. Chem. Theory Comput.* **12**, 405–413 (2016).

21. Pronk, S. *et al.* GROMACS 4.5: a high-throughput and highly parallel open source molecular simulation toolkit. *Bioinformatics* **29**, 845–854 (2013).

22. de Jong, D. H. *et al.* Improved Parameters for the Martini Coarse-Grained Protein Force Field. *J. Chem. Theory Comput.* **9**, 687–697 (2013).

23. Wassenaar, T. A., Ingólfsson, H. I., Böckmann, R. A., Tieleman, D. P. & Marrink, S. J. Computational Lipidomics with insane: A Versatile Tool for Generating Custom Membranes for Molecular Simulations. *J. Chem. Theory Comput.* **11**, 2144–2155 (2015).

24. Kollman, P. A. *et al.* Calculating structures and free energies of complex molecules: combining molecular mechanics and continuum models. *Acc. Chem. Res.* **33**, 889–897 (2000).

25. Junmei, W., Tingjun, H. & Xiaojie, X. Recent Advances in Free Energy Calculations with a Combination of Molecular Mechanics and Continuum Models. *Curr. Comput. Aided Drug Des.* **2**, 287–306 (2006).

26. Salomon-Ferrer, R., Case, D. A. & Walker, R. C. An overview of the Amber biomolecular simulation package. *Wiley Interdiscip. Rev. Comput. Mol. Sci.* **3**, 198–210 (2013).

27. Salomon-Ferrer, R., Götz, A. W., Poole, D., Le Grand, S. & Walker, R. C. Routine Microsecond Molecular Dynamics Simulations with AMBER on GPUs. 2. Explicit Solvent Particle Mesh Ewald. *J. Chem. Theory Comput.* **9**, 3878–3888 (2013).

28. Maier, J. A. *et al.* ff14SB: Improving the Accuracy of Protein Side Chain and Backbone Parameters from ff99SB. *J. Chem. Theory Comput.* **11**, 3696–3713 (2015).

29. Joung, I. S. & Cheatham, T. E., 3rd. Determination of alkali and halide monovalent ion parameters for use in explicitly solvated biomolecular simulations. *J. Phys. Chem. B* **112**, 9020–9041 (2008).

30. Darden, T., York, D. & Pedersen, L. Particle mesh Ewald: An N·log(N) method for Ewald sums in large systems. *J. Chem. Phys.* **98**, 10089–10092 (1993).

31. Berendsen, H. J. C., Postma, J. P. M., van Gunsteren, W. F., DiNola, A. & Haak, J. R. Molecular dynamics with coupling to an external bath. *J. Chem. Phys.* **81**, 3684–3690 (1984).

32. Mercurio, F. A., Scaloni, A., Caira, S. & Leone, M. The antimicrobial peptides casocidins I and II: Solution structural studies in water and different membrane-mimetic environments. *Peptides* **114**, 50–58 (2019).

33. Shalev, D. E., Rotem, S., Fish, A. & Mor, A. Consequences of N-Acylation on Structure and Membrane Binding Properties of Dermaseptin Derivative K4-S4-(1-13)*. *J. Biol. Chem.* **281**, 9432–9438 (2006).

34. Wu, J.-M. *et al.* Structure and function of a custom anticancer peptide, CB1a. *Peptides* **30**, 839–848 (2009).

35. Dubovskii, P. V. *et al.* Three-dimensional structure/hydrophobicity of latarcins specifies their mode of membrane activity. *Biochemistry* **47**, 3525–3533 (2008).

36. Lee, K. H. *et al.* Interactions between the plasma membrane and the antimicrobial peptide HP (2-20) and its analogues derived from Helicobacter pylori. *Biochem. J* **394**, 105–114 (2006).

37. Bourbigot, S. *et al.* Antimicrobial peptide RP-1 structure and interactions with anionic versus zwitterionic micelles. *Biopolymers* **91**, 1–13 (2009).

38. Wu, P.-S., Lai, S.-J., Fung, K.-M. & Tseng, T.-S. Characterization of the structure–function relationship of a novel salt-resistant antimicrobial peptide, RR12. *RSC Adv.* **10**, 23624–23631 (2020).

39. Dubovskii, P. V. *et al.* Novel lynx spider toxin shares common molecular architecture with defense peptides from frog skin. *FEBS J.* **278**, 4382–4393 (2011).

40. Cardoso, M. H. *et al.* A Computationally Designed Peptide Derived from Escherichia coli as a Potential Drug Template for Antibacterial and Antibiofilm Therapies. *ACS Infect Dis* **4**, 1727–1736 (2018).

41. Gomes, I. P. *et al.* Membrane interactions of the anuran antimicrobial peptide HSP1-NH2: Different aspects of the association to anionic and zwitterionic biomimetic systems. *Biochim. Biophys. Acta Biomembr.* **1863**, 183449 (2021).

42. Irazazabal, L. N. *et al.* Fast and potent bactericidal membrane lytic activity of PaDBS1R1, a novel cationic antimicrobial peptide. *Biochim. Biophys. Acta Biomembr.* **1861**, 178–190 (2019).

43. Pillong, M. *et al.* Rational Design of Membrane-Pore-Forming Peptides. *Small* **13**, 1701316 (2017).

44. Xiao, Y. *et al.* Structure-activity relationships of fowlicidin-1, a cathelicidin antimicrobial peptide in chicken. *FEBS J.* **273**, 2581–2593 (2006).

45. Cantini, F. *et al.* Effect of positive charges in the structural interaction of crabrolin isoforms with lipopolysaccharide. *J. Pept. Sci.* **26**, e3271 (2020).

46. Mariano, G. H. *et al.* Characterization of novel human intragenic antimicrobial peptides, incorporation and release studies from ureasil-polyether hybrid matrix. *Mater. Sci. Eng. C Mater. Biol. Appl.* **119**, 111581 (2021).

47. Dubovskii, P. V. *et al.* Spatial structure and activity mechanism of a novel spider antimicrobial peptide. *Biochemistry* **45**, 10759–10767 (2006).

48. Zhu, S., Aumelas, A. & Gao, B. Convergent evolution-guided design of antimicrobial

peptides derived from influenza A virus hemagglutinin. *J. Med. Chem.* **54**, 1091–1095 (2011).

49. Lee, K. *et al.* Antibiotic activity and structural analysis of the scorpion-derived antimicrobial peptide IsCT and its analogs. *Biochem. Biophys. Res. Commun.* **323**, 712–719 (2004).

50. Subasinghage, A. P., O'Flynn, D., Conlon, J. M. & Hewage, C. M. Conformational and membrane interaction studies of the antimicrobial peptide alyteserin-1c and its analogue [E4K]alyteserin-1c. *Biochim. Biophys. Acta* **1808**, 1975–1984 (2011).

51. Bommineni, Y. R. *et al.* Fowlicidin-3 is an alpha-helical cationic host defense peptide with potent antibacterial and lipopolysaccharide-neutralizing activities. *FEBS J.* **274**, 418–428 (2007).

52. Batista Martins, D. *et al.* Protonectin peptides target lipids, act at the interface and selectively kill metastatic breast cancer cells while preserving morphological integrity. *J. Colloid Interface Sci.* **601**, 517–530 (2021).

53. Schibli, D. J., Hwang, P. M. & Vogel, H. J. Structure of the antimicrobial peptide tritrpticin bound to micelles: a distinct membrane-bound peptide fold. *Biochemistry* **38**, 16749–16755 (1999).

54. Ovchinnikov, K. V. *et al.* Defining the Structure and Receptor Binding Domain of the Leaderless Bacteriocin LsbB*. *J. Biol. Chem.* **289**, 23838–23845 (2014).

55. Bhunia, A., Ramamoorthy, A. & Bhattacharjya, S. Helical hairpin structure of a potent antimicrobial peptide MSI-594 in lipopolysaccharide micelles by NMR spectroscopy. *Chemistry* **15**, 2036–2040 (2009).

56. Xiao, Y. *et al.* The central kink region of fowlicidin-2, an alpha-helical host defense

peptide, is critically involved in bacterial killing and endotoxin neutralization. *J. Innate Immun.* **1**, 268–280 (2009).

57. Petit, V. W. *et al.* A hemocyanin-derived antimicrobial peptide from the penaeid shrimp adopts an alpha-helical structure that specifically permeabilizes fungal membranes. *Biochim. Biophys. Acta* **1860**, 557–568 (2016).

58. Tseng, T.-S., Tsai, K.-C. & Chen, C. Characterizing the structure-function relationship reveals the mode of action of a novel antimicrobial peptide, P1, from jumper ant Myrmecia pilosula. *Mol. Biosyst.* **13**, 1193–1201 (2017).

59. Uteng, M. *et al.* Three-dimensional structure in lipid micelles of the pediocin-like antimicrobial peptide sakacin P and a sakacin P variant that is structurally stabilized by an inserted C-terminal disulfide bridge. *Biochemistry* **42**, 11417–11426 (2003).

60. Lee, J. T. Y., Wang, G., Tam, Y. T. & Tam, C. Membrane-Active Epithelial Keratin 6A Fragments (KAMPs) Are Unique Human Antimicrobial Peptides with a Non-αβ Structure. *Front. Microbiol.* **7**, 1799 (2016).

61. Sprules, T., Kawulka, K. E., Gibbs, A. C., Wishart, D. S. & Vederas, J. C. NMR solution structure of the precursor for carnobacteriocin B2, an antimicrobial peptide from Carnobacterium piscicola. *Eur. J. Biochem.* **271**, 1748–1756 (2004).
