# Supplementary material for: Helical structure motifs made searchable for functional peptide design
Source: Nat Commun. 2022 Jan 10;13:102. doi: 10.1038/s41467-021-27655-0 (PMC8748493; doi:10.1038/s41467-021-27655-0)
Supplement: Supplementary file 3 — Description of Additional Supplementary Files [file 41467_2021_27655_MOESM3_ESM.pdf]

Description of Additional Supplementary Files

File Name: Supplementary Data 1
Description: All the full helices deposited in TP-DB database have included in the Supplementary Data 1 (file name: Supplementary_Data_1.zip) shared in the Zenodo repository (https://doi.org/10.5281/zenodo.56532 87). Each row of this table contains the identifier of the sequence comprising the structural source (via PDB accession codes), chain ID, type of helix, the location and range of the helix (noted by the residue ID), the sequence itself, helical propensity, contact number, and the hyperlink to the structure of the helix in the PDB format. See the README file shared in the Zenodo repository for details.

File Name: Supplementary Movie 1
Description: All the three AMPs were first created as fully extended AMPs and they were then simulated in an NPT ensemble for 50 ns in the absence of lipids. The resulting snapshots of the AMPs were clustered into 5 groups based on their structural similarity using the "clustering" plug-in of the VMD software4. The representative conformation from the biggest cluster in each of the three AMPs was selected as the initial conformation to be simulated from time zero in an NPT ensemble in the presence of the already equilibrated PC/PG (3:1) membrane. At time zero, as seen in the video, all the peptides have been folded into a helical shape in the CHARMM36 forcefield2,3. Within 1 microsecond, the tryptophan residues in W3_p1 had all three Trp and W3_p2 had two Trp (W3 and W6) inserted into the membrane (especially after 800 ns), while only one Trp (W3) in W3_db5 had been inserted into the membrane. (Supplementary_Movie_1.mov of 18.9MB) and Zenodo repository https://doi.org/10.5281/zenodo.565328 7 (Supplementary_Movie_1.mp4 file of 76.3MB with higher resolution).
