# Supplementary material for: Helical structure motifs made searchable for functional peptide design
Source: Nat Commun. 2022 Jan 10;13:102. doi: 10.1038/s41467-021-27655-0 (PMC8748493; doi:10.1038/s41467-021-27655-0)
Supplement: Supplementary file 6 — Reporting Summary [file 41467_2021_27655_MOESM6_ESM.pdf]

nature portfolio

Corresponding author(s): Chung-Yu Lan*, Hua-Wen Fu* and Lee-Wei Yang*

Last updated by author(s): Nov 12, 2021

Reporting Summary

Nature Portfolio wishes to improve the reproducibility of the work that we publish. This form provides structure for consistency and transparency in reporting. For further information on Nature Portfolio policies, see our Editorial Policies and the Editorial Policy Checklist.

Statistics

For all statistical analyses, confirm that the following items are present in the figure legend, table legend, main text, or Methods section.

n/a	Confirmed	
[ ]	[x]	The exact sample size (*n*) for each experimental group/condition, given as a discrete number and unit of measurement
[ ]	[x]	A statement on whether measurements were taken from distinct samples or whether the same sample was measured repeatedly
[ ]	[x]	The statistical test(s) used AND whether they are one- or two-sided<br>*Only common tests should be described solely by name; describe more complex techniques in the Methods section.*
[x]	[ ]	A description of all covariates tested
[x]	[ ]	A description of any assumptions or corrections, such as tests of normality and adjustment for multiple comparisons
[ ]	[x]	A full description of the statistical parameters including central tendency (e.g. means) or other basic estimates (e.g. regression coefficient) AND variation (e.g. standard deviation) or associated estimates of uncertainty (e.g. confidence intervals)
[ ]	[x]	For null hypothesis testing, the test statistic (e.g. *F*, *t*, *r*) with confidence intervals, effect sizes, degrees of freedom and *P* value noted<br>*Give P values as exact values whenever suitable.*
[x]	[ ]	For Bayesian analysis, information on the choice of priors and Markov chain Monte Carlo settings
[x]	[ ]	For hierarchical and complex designs, identification of the appropriate level for tests and full reporting of outcomes
[ ]	[x]	Estimates of effect sizes (e.g. Cohen's *d*, Pearson's *r*), indicating how they were calculated

Our web collection on statistics for biologists contains articles on many of the points above.

Software and code

Policy information about availability of computer code

Data collection	The helical peptides are parsed from ~130,000 PDB files; the in-house python code that is used to extract the helical peptides from a PDB file can be found in the "data_collection" folder in https://github.com/emmanuelsalawu/tp-db. The data themselves can be found in an online repository on Zenodo at https://doi.org/10.5281/zenodo.5653287.
Data analysis	All MD simulations were performed using either NAMD package (v2.9) or OpenMM with CHARMM36 forcefields. Simulations were performed for 100ns and 1 s in the case of TP-DB peptides and AMPs, respectively, at a time step of 2 fs. RATTLE and SETTLE algorithms are applied to constrain hydrogen atoms in peptides and waters. MS Excel v16.47 and SigmaPlot v12.0 were used for drawing the error bars and for calculating the test statistics and the corresponding p-values for the Student's t tests. Custom codes written in Python and C++ were used for calculating helical propensity, residue contacts, and other metrics. We make the custom codes freely available online on Zenodo (https://doi.org/10.5281/zenodo.5653287) and on Gighub (https://doi.org/10.5281/zenodo.5675428).

For manuscripts utilizing custom algorithms or software that are central to the research but not yet described in published literature, software must be made available to editors and reviewers. We strongly encourage code deposition in a community repository (e.g. GitHub). See the Nature Portfolio guidelines for submitting code & software for further information.

Data

Policy information about availability of data

All manuscripts must include a data availability statement. This statement should provide the following information, where applicable:

- Accession codes, unique identifiers, or web links for publicly available datasets
- A description of any restrictions on data availability
- For clinical datasets or third party data, please ensure that the statement adheres to our policy

The full datasets of generated and analyzed helical peptides, both encoded and the original, in the current study are available freely without restrictions in an online repository on Zenodo at https://doi.org/10.5281/zenodo.5653287. For example, all the helices are in the helical_sequences.zip in FASTA format. The header of each of the sequences in FASTA format is a unique identifier for that sequence and is constituted by PDB-ID, chain-ID, type-of-helix, starting position, ending position (e.g., >2b29_A_1_8_17).

All the codes used for TP-DB are available at GitHub (https://doi.org/10.5281/zenodo.5675428) and Zenodo (https://doi.org/10.5281/zenodo.5653287).

The main data supporting the findings of this study are available in the supplementary information, Source Data file, and other Supplementary Data including the Supplementary Movie 1 and Supplementary Data 1. Supplementary Movie 1 describes the insertion process of AMPs into the POPC/POPG (3:1) membrane observed in MD simulations and is available in both Nat. Commun. website (.mov file of 18.9MB) and Zenodo repository (.mp4 file of 76.3MB with higher resolution). Supplementary Data 1 containing all the helices used in this study is made available in the Zenodo repository (file name "Supplementary_Data_1.zip"). The corresponding descriptions of each helix, including the sequences, their sources in PDB and hyperlinks to their structure files (see the README.txt file on the Zenodo repository https://doi.org/10.5281/zenodo.5653287).

The raw data associated with the figures referred in this study are provided in separate sheets within the "Source Data file" as listed below:
Figure 2a, Figure 2b, Figure 4 and Figure 5 in main-text;
Supplementary Figure 2a, Supplementary Figure 2b, Supplementary Figure 3, Supplementary Figure 5b and Supplementary Figure 7 in Supplementary materials.

The third-party image shown in Supplementary Figure 4 is reproduced from the review article (Marston, A. L. "Shugoshins: tension-sensitive pericentromeric adaptors safeguarding chromosome segregation." Mol. Cell. Biol. 35, 634–648 (2015)), with written permission obtained from the publisher through the Copyright Clearance Center (https://marketplace.copyright.com/rs-ui-web/mp).

Field-specific reporting

Please select the one below that is the best fit for your research. If you are not sure, read the appropriate sections before making your selection.

☒ Life sciences ☐ Behavioural & social sciences ☐ Ecological, evolutionary & environmental sciences

For a reference copy of the document with all sections, see nature.com/documents/nr-reporting-summary-flat.pdf

Life sciences study design

All studies must disclose on these points even when the disclosure is negative.

Sample size	Conventional triplicate repeats and p-value=0.05 were used to draw conclusion for statistical difference between groups. From the operational consistency and the small p-values obtained between compared groups of interest (e.g. Figure 4), we consider that such conventional usage of three repeats and p-value cutoff suffice our research purposes. Majority MIC in pentaplicate biological repeats (at least 3 out 5) were used for quantify AMPs' efficacy, which is a relatively cautious measure as compared to the field standard (Beckloff, N., et al., 2007; https://doi.org/10.1128/AAC.00208-07). The pentaplicate repeats were done twice by different research staffs while giving consistent results. In our MHC tests, triplicate (n = 3) tests can already help us draw statistical conclusions in most of the tested AMP concentrations.
Data exclusions	No data were excluded from the analyses.
Replication	All attempts at replication were successful and results can be reproduced independently by different research staffs (see above).
Randomization	This has little relevance to our study because the query of "WxxWxxW" pattern only resulted in 11 unique sequences. Within the 11, W3-db5 is the peptide having the highest positive charge, which is the only peptide predicted to be effective before the experimental verification (and therefore no bias of choice could be made). For the two negative controls, W3_n1 and W3_n2, we indeed could make other choices but we chose them at random. For the Sgo1-PP2A study, we computationally tested all the peptide blockers found from TP-DB; no bias of choice could be made.
Blinding	linding was only partially done. In the test of the negative controls of W3_n1 and W3_n2, the experimental staffs were not told those are negative controls before obtaining the results. However, in rest of the experiments, the people who prepared the samples were the people who tested the samples and obtained the results so they knew which samples were which.

Reporting for specific materials, systems and methods

We require information from authors about some types of materials, experimental systems and methods used in many studies. Here, indicate whether each material, system or method listed is relevant to your study. If you are not sure if a list item applies to your research, read the appropriate section before selecting a response.

Materials & experimental systems

n/a	Involved in the study
	☒ Antibodies
☒	Eukaryotic cell lines
☒	Palaeontology and archaeology
☒	Animals and other organisms
	☒ Human research participants
☒	Clinical data
	☒ Dual use research of concern

Methods

n/a	Involved in the study
☒	ChIP-seq
☒	Flow cytometry
☒	MRI-based neuroimaging

Antibodies

Antibodies used

(1) The mouse anti-FLAG M2 antibodies were purchased from Sigma-Aldrich (St Louis, MO, USA) with clone name "M2" and catalog number "F-3165".
(2) The hybridoma culture supernatants containing mouse monoclonal antibody MAb 16F4 were the gifts from Drs. Evanthia Galanis and Ianko D. Iankov at Mayo Clinic, USA. They were generated using bacterial cell derived whole NAP molecule from H. pylori 43504 according to reported study [PMID: 21182995], which is the supplementary reference 15 of the manuscript.
(3) The IgG antibodies were purified from mouse serum are purchased from Sigma-Aldrich (St Louis, MO, USA) with catalog number "I5381".
(4) The horseradish peroxidase-conjugated goat anti-mouse secondary antibodies were purchased from Jackson ImmunoResearch (West Grove, PA, USA) with catalog number "115-035-003".

Validation

(1) The mouse anti-FLAG M2 antibodies are validated by Sigma-Aldrich for their sensitivities (using chemiluminescent detection), purity (using capillary electrophoresis) and specificity (using western blot). The antibody is characterized by its specificity to detect FLAG tag (peptide sequence DYKDDDDK). The novel application of this antibody to detect HP-NAP is shown in the Figure 2 of the main text of manuscript. More relevant citations regarding the binding capability against FLAG sequence can be found in the Sigma-Aldrich website: https://www.sigmaaldrich.com/catalog/product/sigma/f3165?lang=en&region=TW#productDetailSafetyRelatedDocs.

(2) The hybridoma culture supernatant containing mouse monoclonal antibody MAb 16F4 is able to detect HP-NAP of H. pylori strain 26695 by Western blotting as shown in Figure 2 in the report [PMID: 28328957], which is the supplementary reference 64 of the manuscript. In another report [PMID: 22750540], the 16F4 antibody was demonstrated to be able to detect the HP-NAP of H. pylori strain 26695 by both ELISA and Western blotting as shown in Figure 1A and Figure 4B, respectively..

(3) The IgG antibodies are isolated from pooled normal mouse serum by fractionation and ion-exchange chromatography, and they are validated by Sigma-Aldrich for the purity >95% (using SDS-PAGE). The antibodies may be used as a reference antigen in a variety of immunoassays including ELISA [PMID: 9616171], immunoprecipitation assay [PMID: 17586580], etc. The further applications regarding this antibody can be viewed in the Sigma-Aldrich website https://www.sigmaaldrich.com/catalog/product/sigma/i5381?lang=en&region=TW.

(4) The peroxidase-conjugated goat anti-mouse secondary antibodies were purified from antisera by immunoaffinity chromatography using antigens coupled to agarose beads, and the antibodies are used to react with whole molecule mouse IgG in various assays such as ELISA or immunoelectrophoresis. The relevant citation regarding the antibody can be viewed in the online database CiteAb (https://www.citeab.com/antibodies/2036643-115-035-003-peroxidase-affinipure-goat-anti-mouse-ig?utm_campaign=Widget+All+Citations&utm_medium=Widget&utm_source=Jackson+Immunoresearch&utm_term=Jackson+ImmunoResearch).

Human research participants

Policy information about studies involving human research participants

Population characteristics

The blood sample was taken from a 33 year-old healthy Asian male researcher.

Recruitment

The recruitment of this study is suitable for the healthy adults who aged from 25 to 60 without major medical history, blood-related disorders, chronic diseases and bad habits of daily life. However, based on the purpose of security, pregnant or breastfeeding women are excluded in this research. Our blood sample was drawn from a 33 year-old healthy Asian male researcher. Self-selection bias can be possible although we could not find any clinical trial data supporting the race-, age- or gender-dependency on AMPs' efficacy and hemolysis.

Ethics oversight

The hemolysis protocol was pre-approved by the Research Ethics Committee of the National Taiwan University Hospital (approval number: 201810004RINA). The blood collections were performed by trained phlebotomists to confirm the safety of the donors. The relevant informed consents were provided to the donors to obtain their agreement on this experiment prior to the process, and the nutritional supplements as compensation for research participants were also provided after the operation.

Note that full information on the approval of the study protocol must also be provided in the manuscript.

Dual use research of concern

Policy information about [dual use research of concern]

Hazards

Could the accidental, deliberate or reckless misuse of agents or technologies generated in the work, or the application of information presented in the manuscript, pose a threat to:

No	Yes	
☒	☐	Public health
☒	☐	National security
☒	☐	Crops and/or livestock
☒	☐	Ecosystems
☒	☐	Any other significant area

Experiments of concern

Does the work involve any of these experiments of concern:

No	Yes	
☒	☐	Demonstrate how to render a vaccine ineffective
☒	☐	Confer resistance to therapeutically useful antibiotics or antiviral agents
☒	☐	Enhance the virulence of a pathogen or render a nonpathogen virulent
☒	☐	Increase transmissibility of a pathogen
☒	☐	Alter the host range of a pathogen
☒	☐	Enable evasion of diagnostic/detection modalities
☒	☐	Enable the weaponization of a biological agent or toxin
☒	☐	Any other potentially harmful combination of experiments and agents
